# Plasmon–Exciton Strong Coupling in Low-Dimensional Materials: From Fundamentals to Hybrid Nanophotonic Platforms

**DOI:** 10.3390/nano15191463

**Published:** 2025-09-23

**Authors:** Peipei Zhao, Chengxi Lü, Siyi Sun, Fan Wu

**Affiliations:** College of Science, Beijing Forestry University, Beijing 100083, China; zpp20041122@bjfu.edu.cn (P.Z.); lcx6216@bjfu.edu.cn (C.L.); sunsiyi@bjfu.edu.cn (S.S.)

**Keywords:** plasmon, exciton, low-dimensional materials, quantum dots, two-dimensional materials, strong coupling, plexciton

## Abstract

Strong coupling has emerged as a central topic in nanophotonics, offering a powerful platform for light–matter interaction studies and advancing quantum technologies. Low-dimensional materials, such as quantum dots (QDs) and two-dimensional (2D) semiconductors, possess pronounced excitonic resonances, high stability, and size-dependent tunability, making them ideal candidates for achieving strong coupling with plasmonic structures. In this review, we systematically summarize recent progress in plasmon low-dimensional material strong coupling. We first introduce the fundamental principles and experimental methods of plasmon–exciton strong coupling, then highlight representative studies on plasmon–QDs and plasmon–2D material hybrid systems, and finally discuss recent advances in multimode strong coupling. This review will provide a comprehensive overview and offer valuable guidance for future studies in strong coupling.

## 1. Introduction

Strong interactions are not only of fundamental importance in quantum optics [1,2,3] but also hold great promise for advanced applications, including quantum information processing [4,5,6,7], quantum networks [8,9], single-atom lasing [10,11], and single-photon switches [12,13]. These effects originate from the strong coupling between quantum emitters and a single-mode optical field. In the strong coupling regime, photons and excitations in matter cease to be independent eigenstates and instead form hybrid quasiparticles polaritons characterized by Rabi splitting in the energy spectrum [14,15,16] and periodic Rabi oscillations in the time domain [17,18,19]. Traditionally, strong coupling has been realized using high-Q resonant cavities in atomic and solid-state systems, but these approaches typically require stringent conditions, such as ultrahigh vacuum, cryogenic temperatures, and complex fabrication [20,21,22].

**Figure 1:**
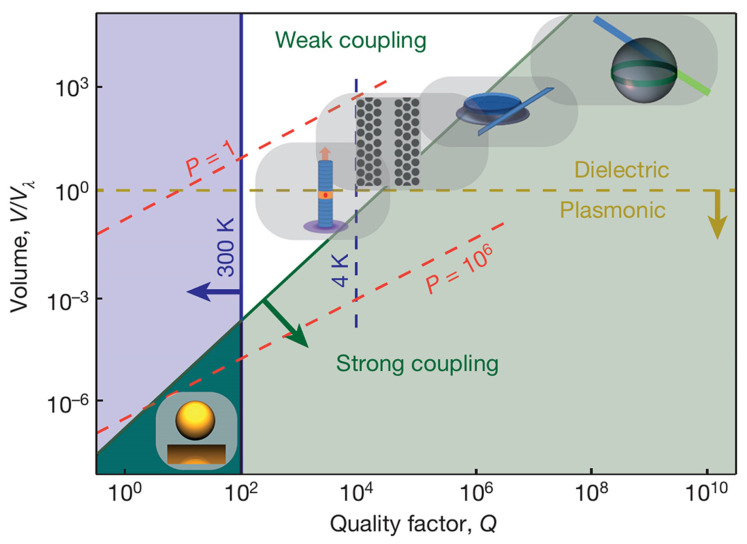
Comparison of coupling conditions between different optical cavities and quantum emitters. Adapted with permission from Ref. [28]. Copyright 2016 Macmillan Publishers Limited.

Surface plasmon polaritons (SPPs) [23,24,25] collective electron oscillations coupled to electromagnetic fields at metal dielectric interfaces offer a powerful alternative for exploring strong coupling at the micro- and nanoscale. Their primary advantage lies in breaking the optical diffraction limit, thereby confining electromagnetic fields within volumes several orders of magnitude smaller than those of dielectric microcavities, as shown in Figure 1. Because the coupling strength between photon and exciton scales inversely with the square root of the mode volume [26,27,28], $g\propto \frac{1}{\sqrt{V_{m}}}$, the deeply subwavelength confinement of plasmonic modes dramatically enhances the interaction strength, ultimately enabling access to the strong coupling regime. Furthermore, unlike dielectric cavities where cryogenic cooling is often required to realize strong coupling [29,30], plasmonic nanostructures support robust strong coupling even at room temperature [28,31], significantly relaxing experimental constraints and lowering the barrier to practical applications; see the comparison in Figure 1. In addition, the resonance frequency of plasmonic modes can be finely tuned through nanostructure geometry, facilitating spectral matching with excitonic transitions and enabling systematic investigations of polariton dispersion [32,33,34].

Strong coupling has been demonstrated with a variety of excitonic systems, including J-aggregates [35,36,37], semiconductor quantum dots (QDs) [38,39], and two-dimensional (2D) materials [40,41,42,43]. These hybrid systems have facilitated investigations into nonlinear polariton dynamics [44,45], chiral interactions [46,47,48], and modified photoluminescence properties [49,50] under strong coupling conditions. While these advances have greatly expanded the scope of physics, quantum effects are strongly dependent on the number of participating excitons. Consequently, approaching the quantum limit of few- or even single-exciton strong coupling is essential. By aggressively compressing plasmonic mode volumes and carefully engineering plasmonic losses, room-temperature single-exciton strong coupling has been demonstrated [16,51,52,53], establishing a foundation for quantum information technologies at the nanoscale.

Early studies in coupling predominantly utilized dye molecules and molecular aggregates [35,36,37]; however, these systems face several limitations. First, their susceptibility to photobleaching and chemical degradation leads to poor stability. Second, their scalability is also severely limited by their poor photostability and the challenges of their dynamic assembly and integration into devices. Furthermore, dye molecules possess a relatively low oscillator strength, which inherently hinders the coupling efficiency with plasmonic modes. The emergence of low-dimensional materials nanostructures with one or more dimensions confined to the nanoscale has opened new opportunities. Quantum confinement in such systems greatly enhances exciton binding energies, stabilizing excitons against dissociation even at room temperature [54,55]. Consequently, low-dimensional materials have become leading candidates for hybrid platforms. A detailed comparison of these materials with their conventional counterparts is provided in Table 1.

As zero-dimensional systems, QDs offer precise size and positional tunability, enabling deterministic placement in plasmonic hot spots to maximize coupling strength [39]. Their high oscillator strength and size-dependent energy levels [63,64], along with further improvements via core shell engineering [65,66,67], make them highly attractive. In parallel, 2D materials, particularly transition metal dichalcogenides (TMDs), boast their own set of advantages. Their strong quantum confinement and weak dielectric shielding result in extremely high exciton binding energy, ensuring their stability even at room temperature [54,68,69]. These materials provide high flexibility for polaritonic system design, owing to their valley degrees of freedom, heterostructure compatibility, and the precise control of bandgap and exciton energies via layer number, temperature, voltage, and magnetic field [62,70,71,72]. In summary, coupling plasmonic nanostructures with low-dimensional excitonic materials represents a promising strategy for engineering hybrid states, with far-reaching implications for both fundamental studies and practical applications in nanophotonics and quantum technologies [73,74,75].

**Figure 2:**
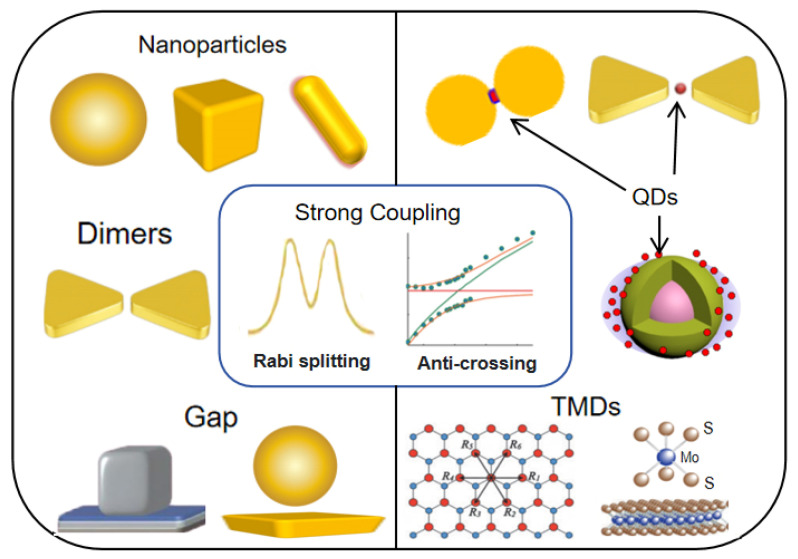
Schematic of in low-dimensional materials strong coupling: the left shows representative plasmonic nanostructures, and the right shows low-dimensional material structure systems. The key characteristics of strong coupling, Rabi splitting, and anti-crossing behavior are highlighted in the center.

However, most existing reviews on plasmon–exciton strong coupling mainly focus on theoretical aspects [76], the choice and design of plasmonic structures [77], or the exploration of coupling phenomena [78]. To the best of our knowledge, there has been no review specifically dedicated to strong coupling from the perspective of low-dimensional excitonic materials. With the widespread application of low-dimensional materials in the design of plasmon strong coupling systems, it is necessary to conduct a systematic review of this research trend. This review focuses on the strong coupling between plasmonic nanostructures and low-dimensional materials, specifically QDs and 2D materials, as depicted in Figure 2. We first outline the fundamental principles and key spectroscopic techniques for exploring strong coupling. Subsequently, we summarize representative advances in plasmon QD hybrid systems and plasmon 2D material platforms, highlighting strategies for achieving few- and single-exciton coupling. Furthermore, we present recent progress in multimode strong coupling involving multiple optical or excitonic modes in low-dimensional materials and discuss emerging approaches for dynamic control and ultrastrong coupling. By systematically covering these developments, this review aims to provide a comprehensive understanding of the current state of the field and offer insights into future directions for nanophotonic and quantum technological applications.

## 2. Basic Principles and Characterization Methods

### 2.1. Basic Principles

In a system, the strong coupling regime is reached when the interaction strength between plasmons and excitons exceeds their average decay rates. Under this condition, the persistent and coherent interaction gives rise to a new hybrid state known as plexcitons [79,80,81]. The fundamental physics of this regime is typically described using several theoretical frameworks, including the Hamiltonian formalism [16,82], the coupled oscillator model [48,83], coupled mode theory [57,84], and the quasi-static approximation [85,86]. Among these, the Hamiltonian method provides a concise and elegant description of the system’s energy eigenstates. By denoting the eigenfrequency of the plasmon mode as $\omega_{p}$, the exciton’s eigenfrequency as $\omega_{x}$, and the coupling strength as g, the Hamiltonian of the lossless system can be expressed as follows [87,88]:

(1) $$\begin{array}{c} H=\hbar \omega_{p}a^{†}a+\hbar \omega_{x}b^{†}b+\hbar g(a^{†}b+ab^{†}) \end{array}$$

Here, $a^{†}(a$) and $b^{†}(b$) are the creation and annihilation operators for plasmons and excitons, respectively.

In realistic systems, both plasmon and exciton modes exhibit finite linewidths due to radiative and nonradiative decay. We can phenomenologically incorporate these losses by introducing a non-Hermitian term [89,90]. Let κ denote the total decay rate of the plasmon mode and γ the total decay rate of the exciton mode. By neglecting quantum noise terms, the effective non-Hermitian Hamiltonian can be written as

(2) $$\begin{array}{c} H_{eff}=\left(\omega_{p}-i\frac{\kappa }{2}\right)a^{†}a+\left(\omega_{x}-i\frac{\gamma }{2}\right)b^{†}b+g(a^{†}b+b^{†}a) \end{array}$$

To solve for the system’s eigenstates, this Hamiltonian can be expressed in a matrix form. On the basis of ($a^{†}$,$b^{†}$), the non-Hermitian Hamiltonian matrix is

(3) $$\begin{array}{c} H_{eff}=\left(\begin{array}{cc} \omega_{p}-i\frac{\kappa }{2} & g\\ g & \omega_{x}-i\frac{\gamma }{2} \end{array}\right) \end{array}$$

Solving the system’s characteristic equation yields two new eigenfrequencies $\omega_{\pm }$, which are given by

(4) $$\;\begin{array}{c} \omega_{\pm }=\frac{1}{2}\left(\omega_{p}+\omega_{x}-i\frac{\kappa +\gamma }{2}\right)\pm \sqrt{g^{2}+\frac{1}{4}{\left(\omega_{p}-\omega_{x}-i\frac{\kappa -\gamma }{2}\right)}^{2}} \end{array}$$

At the resonance condition ($\omega_{p}=\omega_{x}=\omega_{0}$), these eigenfrequencies simplify to

(5) $$\begin{array}{c} \omega_{\pm }=\omega_{0}-i\frac{\kappa +\gamma }{4}\pm \sqrt{g^{2}-\frac{(\kappa -\gamma {)}^{2}}{16}} \end{array}$$

The Rabi splitting, defined as the real part of the spectral splitting, is given by the difference between the real parts of these two eigenfrequencies as

(6) ΩR=2 Re(ω+−ω−)=2 Reg2−(κ−γ)216

On the spectrum, the most direct evidence of strong coupling is the appearance of two distinct resonance peaks. For these two peaks to be clearly resolved, the Rabi splitting must be greater than or at least equal to the average linewidth of the system. This leads to the critical condition for strong coupling:

(7) $$\begin{array}{c} \Omega_{R}\geq \frac{\kappa +\gamma }{2} \end{array}$$

Substituting Formula (7) into Formula (6) yields a clear criterion for strong coupling, namely,

(8) $$\begin{array}{c} g\geq \sqrt{\frac{\kappa^{2}+\gamma^{2}}{8}} \end{array}$$

The two defining hallmarks of the strong coupling regime are energy level splitting and anti-crossing behavior in the center of Figure 2. This splitting is a direct consequence of the coherent coupling between the plasmon and exciton modes, which hybridizes them to form two new eigenstates: the upper plexcitonic branch and the lower plexcitonic branch. As a result, the energies of these new modes are significantly shifted relative to their original, uncoupled states [14,15,16]. The definitive spectroscopic signature of this process is the anti-crossing behavior, which is observed when the energy of the plasmon or exciton is tuned through resonance. Specifically, rather than simply crossing, the energy levels of the two new hybrid modes “repel” each other, creating a distinct avoided crossing feature [48,58,91], as shown in the center of Figure 2. Therefore, the observation of both energy splitting and this characteristic anti-crossing behavior is the conclusive evidence that a system has entered the strong coupling regime.

### 2.2. Characterization Methods

Spectral analysis serves as the cornerstone for characterizing strong coupling, since the pronounced modifications to the system’s energy levels can be directly reflected in the optical spectra [91,92]. Specifically, by examining the resonant features in these spectra, one can determine the energies of the hybridized modes and, critically, extract the Rabi splitting, which remains the most widely used experimental metric for quantifying coupling strength [76,78]. Ultimately, the choice of which spectrum to measure depends on the specific system and its characteristics.

**Figure 3:**
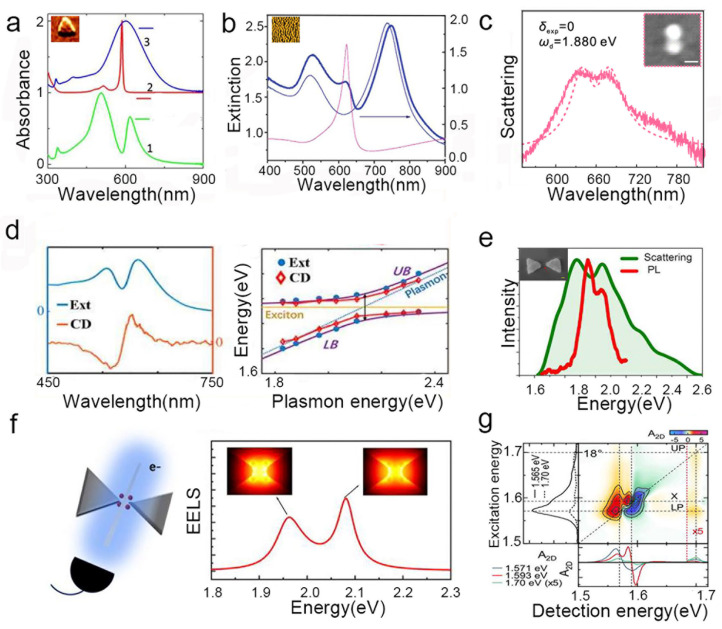
Schematic diagram of the strong coupling characterization method. (**a**) Absorption spectra of silver nanorod colloidal solution (blue), TDBC solution (red), and silver nanorod J-aggregate mixed nanostructured colloidal solution (green). Adapted with permission from Ref. [37]. Copyright 2013 Optical Society of America. (**b**) Extinction spectrum of the hybrid ANR (thick blue line), along with the extinction of the isolated ANR (thin blue line) and the extinction of the J-aggregate on a Au film (magenta line). Adapted with permission from Ref. [93]. Copyright 2007 American Chemical Society. (**c**) Scattering spectra of an individual AuND/MB hybrid with zero detuning. Adapted with permission from Ref. [51]. Copyright The Author(s) 2024. (**d**) Circular dichroism spectra and absorption spectra of the composite exciton system composed of composite metal nanoparticles and chiral J-aggregates. Adapted with permission from Ref. [58]. Copyright 2020 American Chemical Society. (**e**) Dark-field scattering spectrum (green) and photoluminescence spectrum (red) of a device containing one QD, with the scanning transmission electron microscope (STEM) image of the device containing one QD shown in the inset; the QD is pointed to by the red arrow in the butterfly gap. Adapted with permission from Ref. [59]. Copyright The Author(s) 2021. (**f**) Near-field EELS of the silver bowtie nanocavity semiconductor quantum dot system. Adapted with permission from Ref. [97]. Copyright 2024 American Chemical Society. (**g**) Experimental 2D electron energy spectrum of J-aggregates nanoslit array. Adapted with permission from Ref. [18]. Copyright The Author(s) 2023.

In early demonstrations, absorption/extinction spectroscopy was used to analyze solution-based samples where metal nanostructures were coated with dye molecules [35,36]. Figure 3a, for example, shows the absorption spectrum of a strongly coupled system consisting of metal nanoparticles and J-aggregates [37], while Figure 3b presents the extinction spectrum of a gold nanorod array strongly coupled to J-aggregates [93]. Both of the systems exhibited a significant spectral mode splitting after coupling. Although these methods provide clear evidence of mode hybridization, they reflect only ensemble-averaged behavior. To overcome the limitations of ensemble measurements, dark-field scattering technology was developed to precisely measure the scattering spectrum of a single coupled nanostructure [83,94]. Figure 3c shows the scattering spectrum of the hybrid system where a single methylene blue molecule (MB) is integrated into a gold nanodimer (AuND), clearly revealing strong coupling at the single-particle level [51]. When studying chiral interactions in the strong coupling regime, circular dichroism (CD) spectra has emerged as a powerful technique [95,96]. Similar to absorption spectra, CD spectra also show mode splitting and anti-crossing behaviors when the system steps to a strong coupling regime. However, the unique CD spectra line shape of the coupled system provides a relative advantage in distinguishing hybrid modes [48,58,91]. Figure 3d demonstrates the spectra and dispersion curve of a composite plexciton system with chiral J-aggregates, where mode splitting and anti-crossing are simultaneously observed in both the CD and absorption spectra [58].

Another indispensable technique is photoluminescence (PL) spectroscopy. Although the mode splitting observed in PL is generally smaller and more challenging to interpret owing to the complex nature of emission processes it provides unique insights into the exciton-specific properties of plexcitons, as this method directly probes excitonic emission while excluding contributions from absorption enhancement, Fano resonances, and other non-strong-coupling effects [59,94,98]. Figure 3e displays both PL and dark-field scattering spectra of a QD-based hybrid device [59], thus highlighting the complementary information accessible through PL. The Rabi splitting observed in the PL spectrum is smaller than that in the scattering spectrum, indicating that emission and scattering processes involve different coupling pathways an aspect that will be further discussed in later sections. In addition to far-field techniques, near-field methods like EELS have been applied to the study of strong coupling, providing a powerful way to obtain high-resolution spatial information about the near-field of the hybrid modes [78,97,99]. Figure 3f shows an EELS map of a bowtie antenna QD system, clearly demonstrating the energy splitting and the spatial distribution of the upper and lower plexciton branches [97].

Beyond spectral analysis, the Rabi oscillation in the time domain is the most direct signature of coherent energy exchange [17,18,19]. Due to the ultrafast nature of coupling with oscillation periods in the tens to hundreds of femtoseconds ultrafast detection techniques are essential for dynamic studies. For instance, Figure 3g shows a 2D coherent electron spectroscopy (2DES) measurement of a gold nanoslit array with a J-aggregate film, revealing the system’s dynamic response [18]. In conclusion, this diverse arsenal of spectroscopic and dynamic detection methods provides a comprehensive and multi-faceted approach to characterizing strong coupling from different dimensions and levels, leading to a deeper understanding of this remarkable physical phenomenon.

## 3. Plasmonic Structure Quantum Dot Strong Coupling

### 3.1. Introduction to Quantum Dots

In optoelectronic materials, when the characteristic size in one or more dimensions is reduced to the nanoscale, atoms and electrons become spatially confined. These systems are known as low-dimensional materials and exhibit unique physical properties, such as exceptional photostability, high electrical conductivity [100,101], and excellent mechanical flexibility [102,103]. Consequently, they have attracted significant attention in various optoelectronic applications [60,104,105].

**Figure 4:**
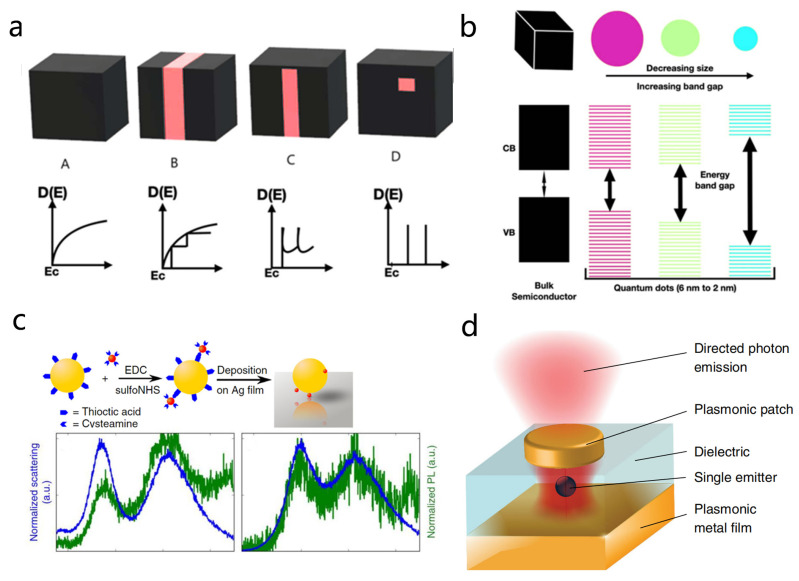
Quantum confinement, size effect, and some plasmon QDs coupling works. (**a**) Schematic illustration of the changes in the density of states: (A) bulk material, (B) 2D, (C) 1D, and (D) 0D. (**b**) Schematic diagram showing energy band structures in bulk material and quantum nanostructure. (**a**,**b**) Adapted with permission from Ref. [107]. Copyright 2023 The Author(s). (**c**) Top: Schematic diagram of the synthesis process for coupled QD and gap plasmon systems. QDs (red) are linked to gold nanoparticles (yellow) via their capping molecules, and the linked components are then deposited on a silver film. Bottom: Measured scattering spectra (blue) and photoluminescence spectra (green) for particles directly on a silver film (left) and with a 5 nm silica spacer between the particles and the silver film (right). Adapted with permission from Ref. [55]. Copyright The Author(s) 2018. (**d**) Schematic diagram of the studied structure, consisting of a single colloidal QD coupled to a plasmonic patch antenna. Adapted with permission from Ref. [110]. Copyright The Author(s) 2020.

QDs are a prototypical class of zero-dimensional (0D) materials, sized from a few to several tens of nanometers with remarkable optoelectronic properties [78,104,106,107,108]. As a result of their size being much smaller than the exciton Bohr radius [109], the motion of electrons and holes is strongly confined in all three dimensions, leading to a pronounced quantum confinement effect [67,107]. This three-dimensional confinement discretizes the electronic states [107] into δ-function-like distributions (Figure 4a), in stark contrast to the continuous energy bands of bulk materials. Moreover, this quantization not only yields sharp absorption and emission spectra but also fundamentally alters their band structure.

Unlike bulk semiconductors with a fixed intrinsic bandgap, the bandgap of QDs is size-tunable, scaling inversely with their size (Figure 4b) [107]. Specifically, smaller QDs experience stronger confinement, resulting in higher transition energies, while larger QDs show weaker confinement and lower energies. As a result, the structural parameters of QDs directly determine their electronic and optical properties [39,107,108,111]. Consequently, this size-dependent tunability provides a powerful means for engineering QD-based optoelectronic devices, making them attractive for emerging technologies such as dynamic quantum light sources [106], tunable nanolasers [112,113], and ultrasensitive biosensing [114], among others [108].

Beyond these applications, the discrete and size-tunable excitonic states of QDs offer an ideal platform for exploring fundamental interactions. Additionally, their strong oscillator strengths make them particularly suitable for coupling with plasmonic nanostructures, motivating extensive research into plasmon QD hybrid systems [76,115]. In such systems, when plasmonic structures and QDs are brought into close proximity, they couple through near-field electromagnetic interactions, which modify the QDs’ radiative dynamics and spectral properties. Depending on the interaction strength, the system is categorized into two regimes: weak coupling and strong coupling [106,116].

Early studies in plasmon QD interactions were primarily in the weak coupling regime [76,115], where the coupling strength was relatively low. In this regime, plasmonic nanostructures primarily modify the QD emission by altering the local density of optical states (LDOS), a phenomenon known as the Purcell effect [117,118]. The fluorescence enhancement of the QD can be quantified by the Purcell factor [119]: $F_{P}=\frac{3}{4\pi^{2}}\left(\frac{Q}{n^{3}}\right)\left(\frac{\lambda_{cav}^{3}}{V_{mode}}\right)$. While plasmonic structures generally have low quality factors (Q), their mode volumes can be far smaller than the wavelength of light, enabling Purcell factors on the order of tens to hundreds. This significantly enhances the radiative rates of QDs, facilitating efficient light emission and control over spontaneous emission. For example, Dhawan et al. demonstrated coupling between a plasmonic patch antenna and a colloidal QD, achieving a 70-fold enhancement in fluorescence efficiency, as shown in Figure 4d [110].

Following the demonstration of strong coupling between plasmonic nanostructures and dye molecules [17,28,37], researchers quickly recognized the distinct advantages of QDs for achieving strong coupling [105,106,110,120]. First, QDs possess large transition dipole moments and narrow linewidths [114,121,122]. Second, they exhibit superior photostability [106,107,122], especially in structures where the internal shell effectively passivates surface defects and presses nonradiative recombination, thereby improving lasing power tolerance by a factor of 3–5 [60,123]. Additionally, QDs can be precisely counted and positioned [15,26,39], providing a natural advantage for achieving strong coupling in few-emitter or even single-emitter systems with plasmonic structures. Capitalizing on these advantages, as illustrated in Figure 4c, Pelton’s team engineered a gold nanoparticle silver film gap plasmonic cavity with CdSe/CdS QDs covalently linked to the gold nanoparticles. They demonstrated strong coupling of single QDs, evidenced by the spectral splitting observed in both the scattering and PL spectra [55].

Collectively, these studies establish QDs as a highly promising platform for building strong coupling systems. Various plasmon QD configurations have been realized, as summarized in Table 2. The following subsections will detail these developments.

### 3.2. Plasmonic Structure Quantum Dot Hybrid Systems

#### 3.2.1. Plasmonic Nanoparticles–Quantum Dot Strong Coupling Systems

**Figure 5:**
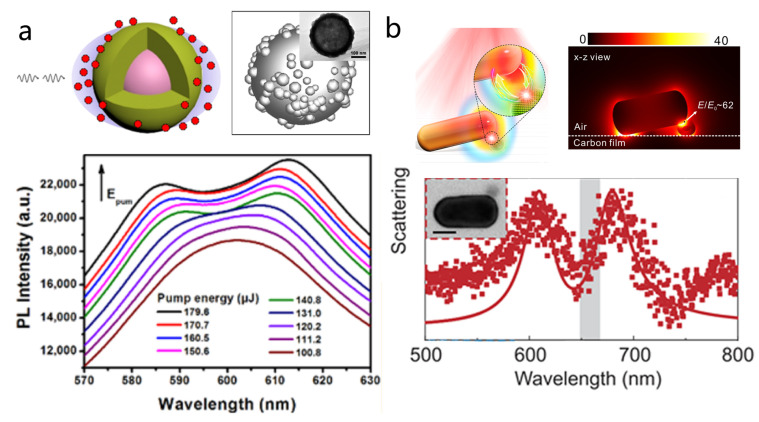
Strong coupling of plasmonic nanoparticles and QDs. (**a**) Strong coupling system of silver nanoshells CdSe/ZnS QDs and their PL spectra at different pump energies. Adapted with permission from Ref. [124]. Copyright 2016 American Chemical Society. (**b**) Left top: Schematic diagram of a wedge-shaped nanogap cavity. Right top: x-z view of the simulated EF distributions of the hybrid Au NR@QD system. Bottom: Normalized scattering spectra of single gold NR@QD hybrids; the inset shows the TEM image of the measured hybrid. Adapted with permission from Ref. [15]. Copyright 2022 American Chemical Society.

Metal nanoparticles supporting localized surface plasmon resonances (LSPRs) represent one of the earliest platforms for constructing plexcitonic systems. Their appeal lies in relatively simple chemical preparation and synthesis [15,28,124,125,126,129]. While early studies have achieved strong coupling with dye molecules or J-aggregates adsorbed onto the nanoparticle surface [28,130,131], doing so with QDs is experimentally more challenging, as it requires precise positioning of QDs around plasmonic hot spots. Yang et al. addressed this by mixing silver nanoshells with a high-concentration CdSe/ZnS QD solution, creating a coupled system between the nanoshells and ensembles of QDs (Figure 5a) [124]. In this configuration, QDs were randomly dispersed around the nanoshells, leading to stochastic spatial coupling with the localized plasmonic near fields. Under non-resonant pulsed laser excitation at room temperature, this ensemble coupling yielded a clear Rabi splitting of approximately 160 meV in the photoluminescence spectrum. The splitting was found to be tunable by excitation photon energy and both QD and nanoparticle concentrations. The work also revealed that rough nanoshell surfaces significantly enhanced local electric fields in the gaps between protrusions, facilitating stronger coupling and enhanced photoluminescence intensity. In a similar colloidal solution-phase approach, Zain et al. demonstrated strong coupling between gold nanorods (AuNRs) and PbS QDs [125]. Unlike the CdSe/ZnS nanoshell system, the AuNR-PbS QD hybrid leveraged the ultrasmall mode volume and tunable LSPR of nanorods, combined with the large dipole moment of PbS QDs. This combination enabled the observation of a more pronounced Rabi splitting of ~231 meV in absorption.

While the work by Zain and Yang et al. demonstrated the feasibility of achieving strong coupling in a colloidal solution with ensembles of QDs, it inherently limited the ability to probe individual emitter–plasmon interactions, which is crucial for fundamental quantum optical effects [28,51]. Precisely positioning a single QD within the nanometric hot spot of an individual plasmonic nanoparticle remains an exceptionally challenging task.

Wang et al. successfully addressed this challenge by integrating a single colloidal QD with a single AuNR, with precise localization verified using transmission electron microscopy (TEM) [15]. Their wedge-shaped nanogap cavity design (Figure 5b) allowed the plasmonic electric field to be tightly confined within the QD region, maximizing the interaction. Using this configuration, they observed a record Rabi splitting of approximately 234 meV, the largest reported to date for a single-QD plasmon system. This deterministic single-QD strong coupling provides a clear demonstration of true quantum hybridization at the single-emitter level, offering a powerful platform for research and applications in quantum optics [132].

#### 3.2.2. Plasmonic Dimers/Gap Structures Quantum Dot Strong Coupling Systems

The above studies highlight the feasibility of strong coupling between nanoparticles and QDs. However, common plasmonic nanostructures like spheres and rods have relatively large resonant mode volumes [55,126,133] and limited diversity in their optical responses [28,127]. To overcome these limitations, composite nanoparticle architectures such as nanoparticle dimers and nanoparticle-on-mirror (NPOM) cavities have emerged. These configurations can further compress the plasmonic mode volume and generate richer hybrid modes, providing a more versatile platform for strong light–matter interactions.

**Figure 6:**
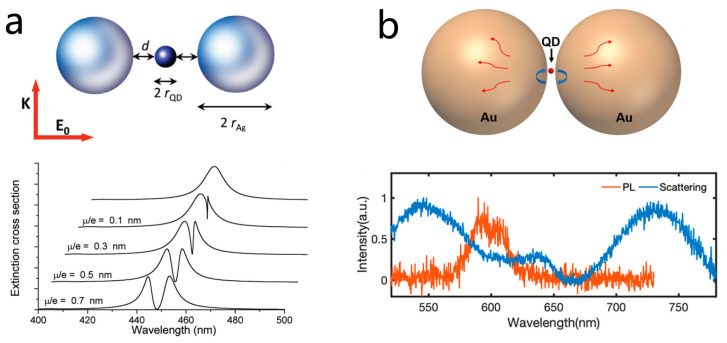
Strong coupling of single QDs with nanosphere dimers. (**a**) Top: Schematic diagram of a nanosphere dimer nanoantenna coupled to a single QD. Bottom: Calculated extinction cross-section as a function of input field wavelength for different quantum dot dipole moments. Adapted with permission from Ref. [134]. Copyright 2010 American Chemical Society. (**b**) Top: Schematic diagram of strong coupling in an Au-QD-Au sandwiched structure. Bottom: Non-polarized dark-field scattering and PL spectra for a single Au nanosphere QD Au nanosphere structure. Adapted with permission from Ref. [26]. Copyright 2020 American Chemical Society.

A key theoretical work was achieved in 2010 when Ferdinando et al. predicted through rigorous scattering calculations that a single quantum emitter placed in the nanogap of a metallic sphere dimer could exhibit vacuum Rabi splitting (Figure 6a) [134]. This work highlighted the immense potential for achieving strong coupling in a hybrid single QD-sphere dimer system. Experimentally realizing such a system, however, remained a formidable challenge due to the stringent requirements of low plasmonic damping, an ultrasmall mode volume, and precise spectral overlap.

In 2020, Zhao et al. demonstrated that strong coupling metal semiconductor nanostructures could be constructed using colloidal assembly techniques [26]. By assembling gold nanospheres with colloidal QDs, they created sandwiched Au nanosphere QD Au nanosphere structures. By tuning the size of the QDs and the assembly conditions, they were able to control the mode volume of the plasmonic nanocavity. As the interparticle gap decreased, clear Rabi splitting emerged in both the dark-field scattering and fluorescence spectra of individual nanostructures, with splitting energies reaching up to 185 meV, as shown in Figure 6b. Crucially, theoretical simulations revealed that this strong coupling originated not from the conventional dipolar resonance but from a higher-order octupolar plasmonic mode of the Au dimer.

While spherical nanoparticle dimers can achieve strong coupling through higher-order modes, their field enhancement is constrained by geometry. In dimer systems, the degree of field localization is largely dictated by the gap size and particle curvature. As emphasized in optical antenna studies [135], bowtie geometries, with their sharp triangular tips, can generate significantly more confined hot spots under comparable gap conditions [135,136], providing stronger near-field enhancement than spherical dimers. This makes bowtie antennas a more efficient platform for mediating interactions between QDs and light.

**Figure 7:**
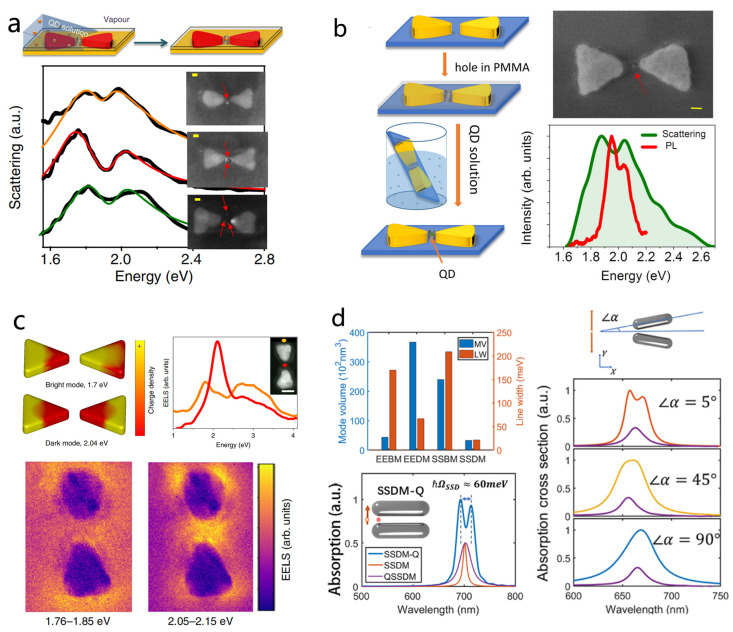
Strong coupling research of QDs with bowtie antennas/nanorod dimers. (**a**) Top: Schematic diagram of a two-step lithography process to create holes in the center of a bowtie structure and the interfacial capillary force-assisted method to drive QDs into the holes. Bottom: Scattering spectra of bowties with (from top to bottom) one, two, and three QDs in the gap, respectively. All spectra show a transparency dip due to Rabi splitting. Insets show the scanning electron microscope (SEM) images of the bowties. The positions of the QDs are marked by red arrows. Adapted with permission from Ref. [39]. Copyright The Author(s) 2016. (**b**) Left: Schematic diagram of the fabrication process for trapping a QD within a plasmonic bowtie. Right top: Scanning TEM image of a device containing one QD. The red arrow points to the QD in the bowtie gap. Right bottom: Dark-field scattering spectrum (green) and PL spectrum (red) of a device containing one QD. Adapted with permission from Ref. [59]. Copyright The Author(s) 2021. (**c**) Left top: Simulated charge density maps for bright and dark dipole modes at the indicated energies. Right top: EELS of a bare bowtie structure measured at two positions indicated by matching colored dots in the inset, which is a high-angle annular dark-field STEM image of the device. Bottom: Experimental EELS maps for bright (left) and dark (right) modes constructed within the indicated energy range. Adapted with permission from Ref. [99]. Copyright The Author(s) 2020. (**d**) Left top: Mode volume and linewidth for end-to-end bright mode, end-to-end dark mode, side-to-side bright mode, and side-to-side dark mode. Left bottom: Absorption spectra of SSDM-Q/SSDM/QSSDM. Right top: Structural diagram of the adjusted side-to-side nanorod. Right bottom: Absorption cross sections of the adjusted SSND-QD coupling systems with different symmetry (red, yellow, and blue lines). The purple lines represent the absorption spectra of the QDs corresponding to different adjusted SSND configurations. Adapted with permission from Ref. [121]. Copyright 2025 Optica Publishing Group.

In 2016, Haran’s group reported a pioneering method for constructing bowtie QD hybrid nanocavities capable of reaching the verge of strong coupling [39]. Their key innovation was positioning semiconductor QDs directly into the nanogaps of silver bowtie antennas. Instead of relying on complex chemical surface modifications, they employed interfacial capillary forces to drive QDs into photolithographically defined nanoholes inside the bowtie gaps (Figure 7a). This effective strategy enabled deterministic placement of one to several QDs within the cavity hot spots, with the number of emitters directly counted. Using this approach, the team observed a distinct transparency dip in the scattering spectra of single bowties, indicative of vacuum Rabi splitting. For single-QD loading, coupling rates as high as 120 meV were recorded, bringing the bowtie QD system close to the strong coupling regime.

Building on their deterministic single-QD location strategy, Haran’s team further investigated the optical characteristics of single-QD coupling in bowtie plasmonic antennas. They observed that the Rabi splitting in PL spectra was consistently smaller than that in scattering spectra (Figure 7b), a phenomenon also reported in other systems but not explained by the conventional two-level Jaynes Cummings model [59]. To account for this, the team introduced an extended model incorporating QD dark states, treating each QD as a three-level system. In this framework, dark states, with long lifetimes and weak coupling, indirectly narrow the PL emission, while scattering spectra predominantly reflect bright-state–plasmon interactions. Hanbury Brown Twiss interferometry at the single-QD level confirmed non-classical emission from 1–3 QDs, and numerical simulations successfully reproduced the experimental trends. This work systematically demonstrated the crucial role of QD dark states in shaping PL spectra in plasmonic cavity QD strong coupling systems.

Plasmonic dimers not only enable ultrasmall mode volumes but also support a richer set of plasmonic resonance modes. In particular, dark plasmon modes offer advantages such as long lifetimes and low radiative losses, making them attractive for strong coupling. However, due to their sub-radiant nature, dark modes cannot be directly excited or detected using conventional far-field optical techniques [99,121,137,138]. Previous studies on strong coupling have predominantly focused on bright modes, and experimental evidence of strong coupling between dark modes and quantum emitters, especially at the single-nanostructure level, has been lacking.

In 2020, Haran et al. directly demonstrated that dark modes of single plasmonic bowties can interact with a few quantum emitters by employing EELS [99]. Unlike conventional optical methods, EELS can probe the near-field electromagnetic distribution of nanostructures with nanometer spatial resolution and simultaneously excite both bright and dark modes (Figure 7c). Using this technique, the team observed vacuum Rabi splitting between the dark mode of silver bowtie nanostructures and QDs at the single-particle level, with a coupling strength of 85 meV. Notably, both experiments and simulations revealed that the strongest electric field of the dark mode is located at the periphery of the bowtie gap rather than at its center, revising the traditional view that the gap center hosts the maximum field.

Fan Wu and Wei Zhang further systematically investigated how the characteristics of dark modes depend on the configuration of metallic nanodimers [121]. They found that variations in dimer geometry significantly influence the mode volume, field distribution, and lifetime of the dimer’s dark modes (Figure 7d). By exploiting these configuration-dependent properties, the team reduced radiative losses and achieved narrower linewidths and longer photon lifetimes, facilitating strong coupling with a single quantum emitter. Furthermore, by introducing symmetry breaking, they demonstrated dynamic switching of a single structure across three distinct coupling states, each exhibiting unique optical properties in both the time and frequency domains. This study highlights the critical role of dimer geometry in tailoring dark-mode interactions and provides a low-loss platform for tunable quantum photonic devices.

Despite these advances, achieving robust single-emitter coupling remains challenging due to the strong spatial inhomogeneity of plasmonic dimer fields. To overcome this, Xiulai Xu and co-workers integrated a bowtie nanocavity with a one-dimensional photonic crystal substrate, which rendered the nanogap field more uniform and stable [126]. Using a single CdSe/ZnS QD, they realized room-temperature strong coupling with a Rabi splitting of 170 meV and a five-fold reduction in photon emission lifetime, providing a practical route toward solid-state cavity QED systems.

**Figure 8:**
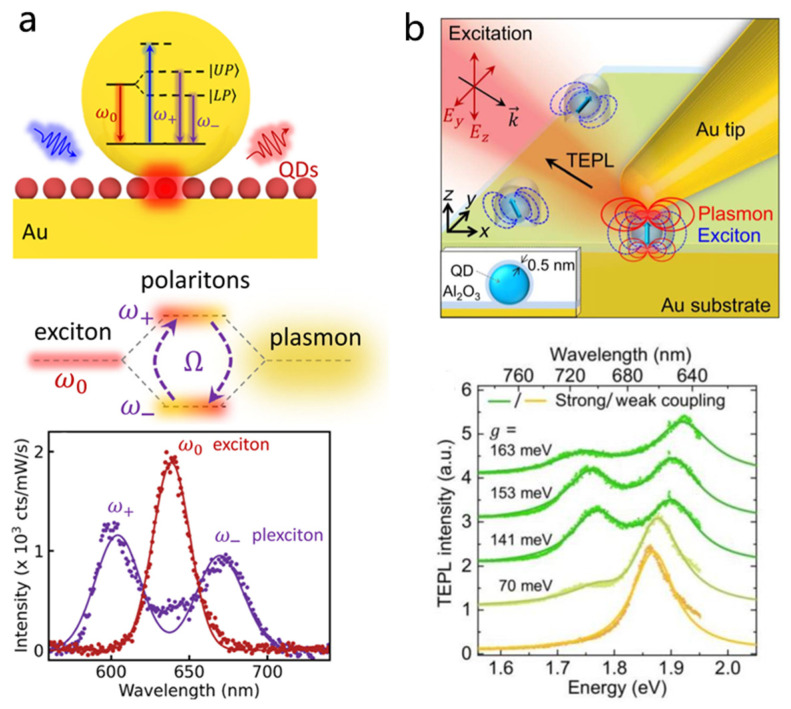
Strong coupling research of QDs with nanogap systems. (**a**) Top: Spontaneous emission process in weak (red) and strong (purple) coupling regimes of NPOM nanocavity coupled to single QD, under non-resonant pumping (blue). Middle: QD and plasmon (cavity) hybridization forms polaritons in a strong coupling regime. Bottom: Photoluminescence spectra of weak (red) and strong (purple) coupled excitons. Adapted with permission from Ref. [131]. Copyright The Author(s) 2024. (**b**) Top: The strongly confined electric fields in a single isolated QD and a tilted Au tip induce coupling between the plasmon and exciton. Bottom: Tip-enhanced photoluminescence spectra of different single QDs with variation in coupling strength g and Rabi frequency. Adapted with permission from Ref. [127]. Copyright 2019 The Authors.

Apart from plasmonic dimers, nanogap systems formed between metallic nanoparticles and metal films NPOM configuration offer an attractive platform for plasmon QD strong coupling due to their simple geometry and reproducible fabrication [139]. Importantly, NPOM cavities support highly confined and uniform gap modes with extremely small mode volumes and strong field enhancement [28,140,141]. Compared to the dimeric structure, the NPOM structure is more conducive to the positioning of quantum dots and the formation of a stable hybrid system. As shown in Figure 8a, Baumberg et al. developed a robust method to produce NPOM nanocavities integrating CdSe/CdS QDs, achieving a high fabrication yield (~74%) of room-temperature single-QD strong coupling with an average Rabi splitting of ~200 meV and even enabling electrically driven exciton–plasmon interactions [131].

Similar to the NPOM configuration, tip-on-film architectures feature highly localized optical fields but provide greater tunability, enabling sub-nanometer control of the gap and coupling strength. Raschke et al. demonstrated strong coupling using a gold tapered tip tilted by 35° on a gold film separated by an ${Al}_{2}O_{3}$ spacer (Figure 8b). By precisely positioning a single QD in the tip-film nanogap, they observed a clear double-peaked PL spectrum with a Rabi splitting up to 163 meV. This platform enables sub-nanometer precision dynamic tuning through lateral and vertical manipulation of the tip, thereby modifying the mode volume and coupling strength [127]. Moreover, the experiment revealed that the coupling strength is strongly dependent on the orientation of the QD’s transition dipole moment, highlighting the role of emitter-field alignment in interactions. Overall, these mirror based nanogap architectures, including NPOM and tip-on-film systems, offer significant advantages for studying plasmon QD strong coupling at the single-emitter level by combining strong field confinement with tunable cavity properties.

#### 3.2.3. Other Plasmon QDs Strong Coupling Systems

**Figure 9:**
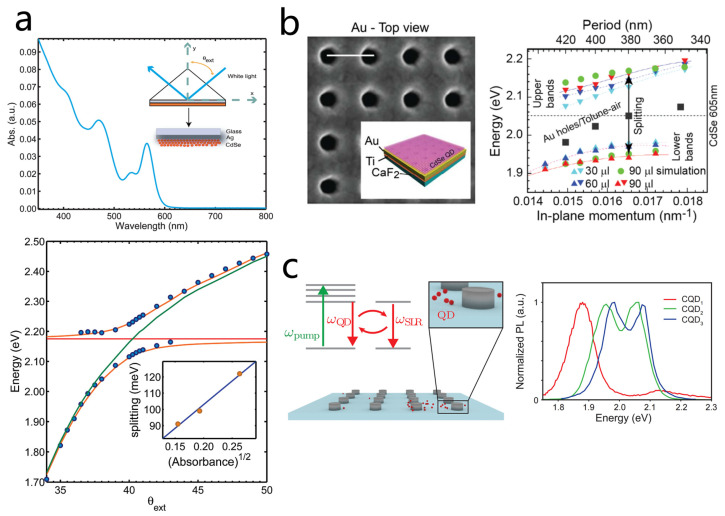
Strong coupling of QDs with other plasmonic structures. (**a**) Top: The normal incidence absorption spectra of CdSe thin films spin-coated on glass slides. The inset shows a schematic diagram of the attenuated total reflection (ATR) experiment. Bottom: Experimental dispersion curve. Adapted with permission from Ref. [111]. Copyright 2010 American Chemical Society. (**b**) Left: SEM image of the hole-based hybrid nanostructure fabricated from top to bottom. Right top: Experimental energy dispersion curves showing the upper and lower bands by varying the amount of CdSe QD solution. Adapted with permission from Ref. [128]. Copyright 2016 American Chemical Society. (**c**) Left: Schematic of CQD plasmonic lattice system. Right: Normalized PL spectra of the CQD samples following introduction into the plasmonic lattice. Adapted with permission from Ref. [116]. Copyright 2020 American Chemical Society.

In the previous sections, we focused on localized plasmonic structures. However, propagating surface plasmon polariton (SPP) modes at extended metal/dielectric interfaces offer an alternative route to strong light–matter interaction [142,143,144]. In fact, the earliest experimental demonstration of plasmon QDs strong coupling was achieved by exciting surface plasmons on a silver film and probing their interaction with the lowest excitonic state of CdSe QDs using the Kretschmann Raether configuration. Attenuated total reflection spectroscopy revealed a Rabi splitting of about 112 meV together with pronounced anti-crossing behavior (Figure 9a) [111]. This experiment in 2009 by Davis’s team marked the starting point for exploring strong coupling with colloidal QDs, laying the foundation for subsequent advances in hybrid systems. In 2010, the same group further demonstrated that SPP fields could coherently couple excitons of two differently sized QDs via a two-oscillator model [145]. However, the SPPs on a uniform metal film surface cannot directly interact with free-space light fields, further restricting their applicability in exciton SPP strong coupling.

Periodic plasmonic arrays are capable of supporting diverse SPP modes that can be directly excited by free-space light. Owing to their ability to significantly enhance optical responses, such arrays have thus been further employed to realize strong coupling with quantum dots [146]. In 2016, Zaccaria’s group investigated strong coupling between CdSe QDs and SPPs in gold nanohole arrays (Figure 9b) using steady-state and femtosecond transient absorption spectroscopy [128]. They observed Rabi splittings up to 220 meV, confirming the formation of hybrid polariton states. Importantly, the large splitting introduced an electron phonon mismatch that suppressed multiphonon relaxation, thereby extending the hybrid state lifetime. This finding offers guidance for designing long-lived plasmon–exciton devices.

Following the work by Zaccaria et al., Basu et al. explored the realization of stronger interactions on this platform (Figure 9c) [116]. By precisely controlling the concentration of colloidal QDs (CQDs), they observed a complete transition from weak to strong coupling between the QDs and the surface lattice resonance (SLR) of a silver nanoparticle array at room temperature. They found that at low concentrations, the QDs predominantly engage in weak coupling with the localized plasmon modes of individual nanoparticles, exhibiting a significant Purcell effect. In contrast, as the QD concentration increases, the system enters the strong coupling regime, where clear upper and lower polariton branches appear in the photoluminescence spectrum. At extremely high concentrations, a novel third emission peak even emerged, originating from a collective QD emission state mediated by the SLR. The foregoing discussion also indicates that SPP modes can likewise achieve strong coupling with quantum dots. However, compared with localized plasmonic structures, SPPs possess a much larger mode volume, which requires the involvement of a large number of quantum dots to achieve strong coupling. Moreover, SPPs cannot directly interact with free-space light fields, further restricting their applicability in exciton–SPP strong coupling. Consequently, research efforts focusing on strong coupling based on SPPs have gradually declined.

In summary, QDs, with discrete, size-tunable excitonic states and high photostability, provide an ideal platform for plasmon–exciton strong coupling. Plasmon QDs strong coupling has been demonstrated across a variety of architectures, ranging from ensemble solutions to dimers and nanogap structures, allowing precise control over plasmonic mode volume and QDs location. The achievement of single-emitter strong coupling represents a significant breakthrough, enabling deterministic manipulation of light–matter interactions at the quantum emitter level. These explorations have deepened our understanding of light–matter interactions and hold promising applications in quantum optics.

## 4. Strong Coupling Between Plasmons and 2D Materials

### 4.1. Introduction to 2D Materials

Two-dimensional materials are atomically thin crystals only one or a few atomic layers thick derived from layered bulk compounds via weak van der Waals interactions [147,148,149]. Commonly studied 2D materials include graphene, known for its high carrier mobility and broadband interaction [150]; hexagonal boron nitride (hBN), which serves as an ideal dielectric with high optical phonon energies and low losses; and transition metal dichalcogenides (TMDs), black phosphorus [151,152,153], MXene [154,155], and so on. Their reduced dimensionality endows them with exceptional electronic, optical, mechanical, and thermal properties [75,156,157,158], making them highly attractive for applications in optoelectronic and micro-nano devices. For instance, TMDs such as MoS_2_ and WS_2_ possess direct bandgaps, making them efficient for light emission and photodetection [159], while graphene offers ultrahigh carrier mobility and broadband optical absorption, suitable for high-speed photodetectors and modulators [160]. Black phosphorus, with its anisotropic band structure and tunable bandgap, further enriches the optical and electronic landscape of 2D materials [161]. At the device level, these materials have been employed in field-effect transistors, flexible photodetectors, optical modulators, and photovoltaic devices, often exhibiting performance advantages over conventional bulk semiconductors [162,163]. Recent advances have further highlighted the versatility of 2D materials for practical applications. For instance, epitaxially engineered WS_2_/MoS_2_ heterostructures exhibit enhanced photo-response owing to improved band alignment and interfacial charge transfer, paving the way for highly efficient photodetectors and optoelectronic devices [164]. In parallel, defect passivation strategies, such as oxygen treatment of sulfur vacancies in monolayer MoS_2_, have been shown to significantly enhance its piezoelectric properties, thereby enabling scalable self-powered nanosystems for wearable sensors and biomedical monitoring [158]. Furthermore, monolayer MoS_2_ has been demonstrated as a robust platform for neuromorphic devices capable of operating reliably at elevated temperatures, offering promising routes for cognitive computing and edge AI in harsh environments [157]. Coupling 2D materials with microcavities, plasmonic structures, and photonic crystals further amplifies their optical responses, paving the way toward quantum optoelectronics, ultrafast photonics, and integrated micro nano systems [165].

Among 2D materials, TMDs stand out as the most widely explored for strong coupling. TMDs consist of a single layer of transition-metal atoms sandwiched between two chalcogen layers, forming hexagonal or pentagonal lattices with the formula MX_2_ (M = Mo, W, Pd; X = S, Se). Common examples include MoS_2_, MoSe_2_, WS_2_, and WSe_2_ [166]. TMDs possess direct bandgaps in the visible regime, large in-plane dipole moments, strong excitonic resonances with binding energies exceeding hundreds of meV, and intrinsically low optical losses, enabling stable exciton polaritons at room temperature [155,167,168,169,170]. Their atomically flat surfaces, lack of dangling bonds, and mechanical flexibility facilitate integration with plasmonic nanostructures via exfoliation, transfer, or CVD growth [171,172,173,174]. Moreover, their optical responses can be dynamically tuned by gating [175], thermal control [176], or layer thickness [177,178], allowing real-time modulation of coupling strength [69,73,179,180]. These features robust excitons, tunability, low loss, and excellent integrability—make TMDs an ideal material platform for achieving and studying strong coupling, with reported Rabi splittings exceeding 200 meV in optimized nanostructures (see the summary in Table 3). In the following sections, we review strong coupling between metal plasmons and TMDs, categorized according to the plasmonic structure employed.

#### 4.1.1. Plasmonic Nanoparticles TMDs Strong Coupling Systems

In early studies of plasmon–exciton strong coupling with 2D materials, silver and gold nanorods were the first employed among the nanoparticle structures [61,83,181]. Metal nanorods are relatively easy to synthesize with high uniformity and offer a key advantage in that their longitudinal plasmon resonance can be tuned over a wide spectral range simply by adjusting the aspect ratio [85,191]. This tunability, combined with their strong field confinement at the rod ends, has made nanorods one of the most widely used nanostructures in plasmonic research [192,193,194]. Importantly, elongated nanorods can support not only dipolar but also higher-order resonant modes, which typically exhibit lower radiative losses [138,195]. These higher-order modes are particularly advantageous for achieving strong light–matter interactions, as they can simultaneously provide tighter confinement and higher quality factors compared to fundamental dipolar modes.

**Figure 10:**
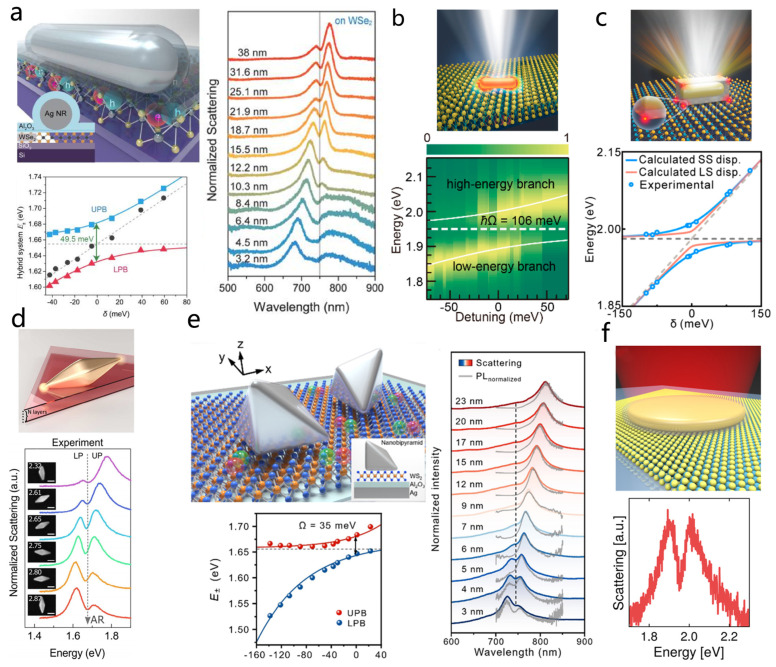
Strong coupling work of nanoparticles with 2D materials. (**a**) Left: Schematic of a single silver nanorod system on a WSe_2_ monolayer. Right: A set of dark-field scattering spectra for the silver nanorod, with increasing alumina coating. Bottom: Energies of the upper polariton branch (UPB) and lower polariton branch (LPB) as a function of detuning. Adapted with permission from Ref. [83]. Copyright 2017 American Chemical Society. (**b**) Top: Schematic of a heterostructure composed of a single gold nanorod coupled with WS_2_. Bottom: Color-coded normalized scattering spectra of heterostructures with different detunings between the plasmon resonance and the exciton. Adapted with permission from Ref. [181]. Copyright 2017 American Chemical Society. (**c**) Top: Schematic of the Au@Ag NCs structure. Bottom: Significant difference between the experimental SS dispersion (blue dots) and theoretical LS dispersion (red line) at different coupling strengths. Adapted with permission from Ref. [61]. Copyright 2024 American Chemical Society. (**d**) Top: Schematic of a gold bipyramid and 2D material structure. Bottom: Scattering spectra of gold bipyramids with different aspect ratios on WSe_2_. Adapted with permission from Ref. [182]. Copyright 2018 American Chemical Society. (**e**) Top left: Schematic showing the anisotropic structure of the NBOM: the gap between the nanobipyramid and the mirror contains WS_2_ and Al_2_O_3_. Right: Coating-dependent dark-field scattering spectra and PL spectra (gray) normalized to the background for 1L-WS_2_-NBOM. Bottom left: Upper and lower polariton energies versus detuning, obtained from fitting the scattering spectra. Adapted with permission from Ref. [40]. Copyright 2025 American Chemical Society. (**f**) Top: Schematic of a single-crystal gold nanoplate with a 2D material structure. Bottom: Scattering spectrum of the coupled system, showing UPB and LPB, with a splitting of approximately 108 meV. Adapted with permission from Ref. [62]. Copyright 2019 American Chemical Society.

Leveraging these advantages, Zheng et al. demonstrated strong coupling between the higher-order Fabry–Pérot mode of a single silver nanorod and excitons in a monolayer WSe_2_ (Figure 10a) [83]. Plexcitons with a Rabi splitting of up to 49.5 meV were observed at room temperature. Notably, the team also achieved in situ measurement of the exciton–plasmon dispersion relation within a single nanocavity by continuously redshifting the plasmon mode through dielectric deposition, eliminating ensemble averaging and sample inhomogeneity (Figure 10a, the left bottom and the right).

Almost simultaneously, Ningsheng Xu and co-workers reported a complementary study in which a single gold nanorod was coupled to a monolayer WS_2_, forming a hybrid system (Figure 10b) [181]. Using the strong excitonic response of WS_2_ and the tight field confinement of a single gold nanorod, the system exhibited giant room-temperature Rabi splitting energies of 91–133 meV with as few as 5–18 excitons. By integrating the hybrid structure into a field-effect transistor, the coupling strength could be actively and reversibly tuned via electrostatic gating, marking the first demonstration of dynamic control of strong coupling in a single plasmonic nanocavity and paving the way for tunable plexcitonic devices. Although the hybrid system based on gold nanorods exhibits broader linewidths compared to the work by Zheng et al., the strong oscillator strength of WS_2_ enhances the coupling strength, making a more pronounced Rabi splitting. Therefore, the choice of excitonic material is a critical factor in the design of strong coupling systems. Following the progress achieved with bare nanorods, coating gold nanorods with a silver shell offers an effective strategy to further compress the plasmonic mode volume and reduce the number of excitons required for strong coupling [16,53]. Building on this method, Zhong et al. realized strong coupling between monolayer WS_2_ excitons and the longitudinal localized surface plasmon mode (LLSPM) of Au@Ag nanocubes (Figure 10c) [61]. Through projected local density of states calculations, they determined the single-exciton coupling strength $g_{0}$ and confirmed that only 7–9 excitons participated in the strong coupling process a significant reduction in the number of excitons compared with previous room-temperature TMDs–plasmon systems [43,83,176,181,183,186]. Their experiments also revealed that spectral splitting (SS) can deviate significantly from intrinsic level splitting (LS), becoming more pronounced at weaker coupling strengths (Figure 10c, bottom). This result challenges the conventional assumption of SS = LS in strong coupling systems and provides new insights into the correct interpretation of Rabi splitting in 2D semiconductor–plasmon hybrids.

Compared to nanorods, bipyramids (BPs) exhibit superior plasmonic characteristics, including narrow resonances and the ability to adopt asymmetric configurations relative to a supporting surface [196,197]. Owing to this asymmetry, typically only one sharp tip of a BP interacts strongly with the excitonic material, while simultaneously forming a tightly confined electromagnetic hotspot. This configuration effectively reduces the mode volume and thereby limits the number of excitons participating in the coupling, which is advantageous for achieving strong light–matter interactions. Michael et al. realized strong coupling using such an asymmetric gold BP placed on WSe_2_ (Figure 10d) [182]. In their design, only the bottom tip of the BP was in contact with the monolayer, while the top tip remained suspended, ensuring highly localized field confinement and efficient overlap. As the aspect ratio decreased, the system displayed characteristic anti-crossing behavior. Building on this structure, Lu et al. designed a silver nanobipyramid-on-mirror (NBOM) structure (Figure 10e), which leveraged two intrinsic advantages of nanobipyramids [40]. First, the anisotropic geometry sustains two orthogonally polarized gap plasmon modes that simultaneously enhance excitation and quantum yield, leading to PL intensities from the NBOM cavity that surpass those of uncoupled excitons in surrounding TMDs by up to $2.1\times 10^{4}$ times. Second, the sharp tips of the nanobipyramid generate a large in-plane vacuum field, resulting in a strong single-exciton coupling strength. With these features, the authors achieved strong coupling between the nanobipyramid and WSe_2_. They also directly observed the characteristic anti-crossing and Rabi splitting signatures in three complementary spectroscopic measurements dark-field scattering, differential reflection, and PL spectra within the same cavity. Analysis of the coupling distribution confirmed that the BP tips dominate the enhancement, and the effective exciton number involved was significantly lower than in conventional cubic nanoparticles.

In addition to these, nanodisks have also achieved strong coupling with TMDs. In 2019, Nicolas Stenger’s team achieved room-temperature strong coupling using the in-plane dipole mode of single-crystal gold nanodisks (as shown in Figure 10f) [62]. The single-crystal gold nanodisks were placed directly on WS_2_, with a 1 nm thick CTAB layer preventing direct contact between the metal and WS_2_ to avoid exciton quenching. The in-plane dipole mode of the gold nanodisks is aligned with the in-plane dipole moment of the WS_2_ excitons, laying the foundation for efficient coupling and verifying that multilayer WS_2_ can significantly enhance the coupling strength. By performing both scattering and reflection spectroscopy, they demonstrated strong coupling with monolayer/few-layer WS_2_ with a Rabi splitting of ~108/175 meV.

#### 4.1.2. Plasmonic Dimer/Nanogap TMD Strong Coupling Systems

**Figure 11:**
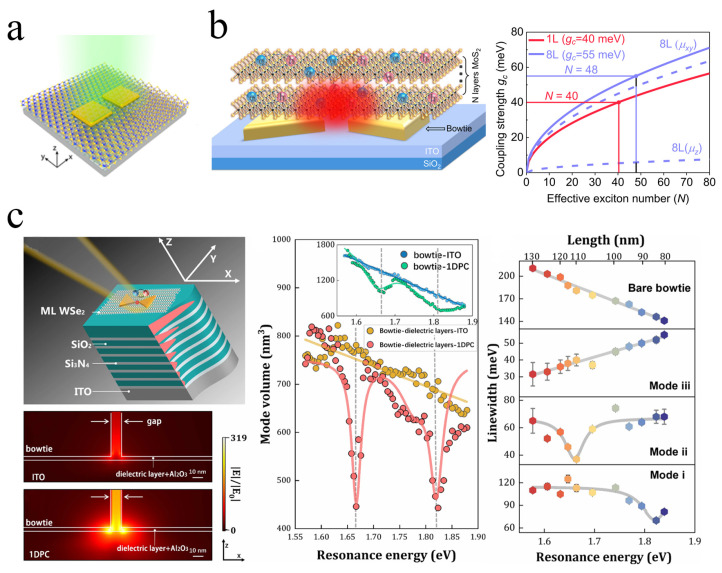
Strong coupling work of plasmonic dimer with 2D materials. (**a**) System of a gold dimer nanoantenna on a monolayer transition metal dichalcogenide. Adapted with permission from Ref. [176]. Copyright 2021 Optical Society of America. (**b**) Left: Schematic of the system with layered MoS_2_ on a single gold bowtie resonator. Right: Coupling strength $g_{c}$ as a function of the effective exciton number (N) for single-layer and eight-layer systems. Adapted with permission from Ref. [183]. Copyright 2022 American Chemical Society. (**c**) Left top: The schematic diagram of the hybrid structure consisting of a single gold bowtie resonator, a 1DPC, and a monolayer WSe_2_. Monolayer WSe_2_ is sandwiched between two cavities. Left Bottom: Electric field distribution of gold bowtie structures on ITO (top) substrate and 1DPC (bottom). Middle: Calculated mode volumes for nanostructures as a function of resonance energy with dielectric layers located on ITO substrates and 1DPC, represented by yellow dots and red dots, respectively. The inset shows the mode volumes for gold bowtie nanostructures on ITO substrates (blue dots) and 1DPC (green dots), respectively. Right: Different plasmon mode line widths versus resonant energy. Adapted with permission from Ref. [184]. Copyright 2025 American Chemical Society.

Dimer structures, owing to their ultrasmall mode volumes, significantly enhance the interaction strength between excitons and confined light fields, making them promising platforms for realizing strong coupling with TMDs [39,80,198,199]. Yu Luo’s team demonstrated few (4.67±0.99) excitons with strong coupling at room temperature by coupling a gold dimer antenna with monolayer WS_2_ (Figure 11a) [176]. Their work revealed the competitive mechanism between exciton number and coupling strength: inserting a 2 nm Al_2_O_3_ spacer increased the number of excitons by tenfold but reduced the coupling strength per exciton by 72%, illustrating the confounding effects of the coupling strength and number of excitons in the observed Rabi splitting in strongly coupled systems. Furthermore, they exploited light-intensity-induced thermal effects to dynamically tune both exciton number and coupling strength, providing a new route for controlling plexcitons at room temperature. The mode volume of square nanodimers can be further increased, for example, by using bowtie antennas.

Xiulai Xu’s team achieved robust strong coupling using a bowtie antenna configuration (Figure 11b) [183]. Combining gold-assisted mechanical exfoliation with a non-destructive wet transfer method, they integrated large-area MoS_2_ monolayers with bowtie antennas. The bowtie mode’s ultrasmall volume and strong in-plane fields reduced the effective number of excitons contributing to the strong coupling. After correcting for exciton transition dipole moments, the exciton numbers were extracted as 40 for a monolayer and 48 for eight layers. More recently, the same group further advanced this system by coupling plasmonic bowtie antennas with Bloch surface waves (BSWs) supported in a one-dimensional photonic crystal (1DPC) (Figure 11c) [184]. The hybrid cavity exhibits a pronounced reduction in mode volume, and the introduction of BSWs also markedly narrows the plasmon linewidth, thereby suppressing plasmonic losses and lowering the threshold for strong coupling. By embedding a monolayer of WSe_2_ within the hybrid cavity, the experiment observed strong coupling with a large Rabi splitting of 186 meV at zero detuning. COM analysis revealed that only about eight excitons participated in the coupling with the plasmonic mode.

**Figure 12:**
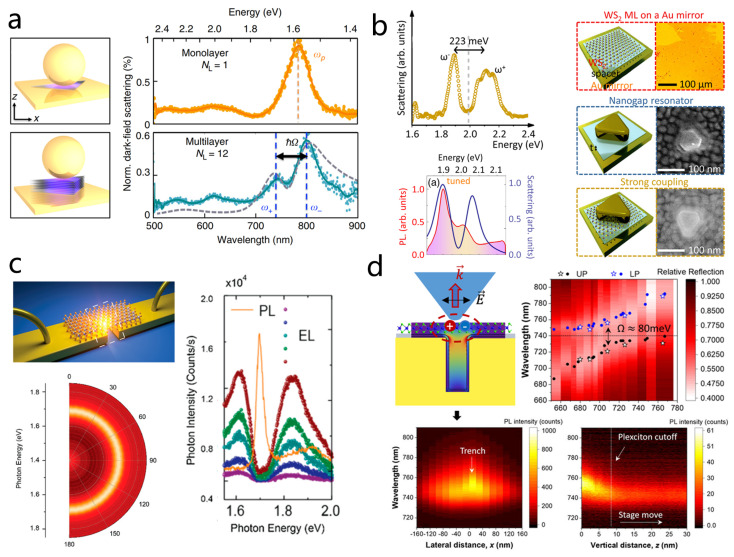
Strong coupling work of metal nanogap with 2D materials. (**a**) Left: Schematic of NPOM cavity encapsulating WSe_2_ flakes for $N_{L}=1,12$. Right: Dark-field scattering of individual NPOMs showing a single-plasmon peak for $N_{L}=1$ WSe_2_ but mode splitting due to strong coupling for multilayer $N_{L}=12$, reproduced by FDTD simulations (dashed line). Adapted with permission from Ref. [185]. Copyright The Author(s) 2017. (**b**) Left top: Scattering spectrum of a WS_2_ monolayer embedded in an NPOM resonator with a spacer thickness t≈1 nm. Right: schematics (optical or SEMs). Left Bottom: PL (red) and scattering (cyan) spectrum for NPOM-WS_2_ systems. Adapted with permission from Ref. [43]. Copyright 2020 American Physical Society. (**c**) Left: schematic of the fabricated TMD hybrid structure on a gap and schematic of coupling connected to electrically driven tunneling in the gap. Left bottom: Normalized polarization-dependent contour map of PL spectra at the nanogap obtained by changing the detecting polarization from 0 to 180°. Right: Measured EL spectra at different biases from 0.8 to 0.9 V with a zero-bias junction conductance of 0.20 $G_{0}$, plotted together with the PL spectrum measured for the WSe_2_ on top. Adapted with permission from Ref. [187]. Copyright 2023 American Chemical Society. (**d**) Left top: Cross-sectional view of the plasmon field intensity enhancement in the bare trench. Right top: Wavelength dependence (vertical axis) of the UPB (black dots/stars) and LPB (red dots/stars) as a function of plasmon resonance wavelength (horizontal axis). Left bottom: PL line scan along the lateral direction measured from a nanotrench at zero detuning with a step size of 20 nm. Right bottom: PL spectra recorded as the probe is retracted from the $z_{0}$ position with a step size of 0.5 nm. Adapted with permission from Ref. [188]. Copyright The Author(s) 2024.

In 2017, Jeremy J. Baumberg’s team embedded mechanically exfoliated WSe_2_ multilayers into the nanogap between a gold nanoparticle and a gold mirror (Figure 12a) [185], forming a plasmonic nanocavity with an NPOM structure. By illuminating the NPOM with blue light, they induced diffusion of gold atoms at the nanoparticle mirror interface, thereby dynamically tuning the plasmon resonance. A clear anti-crossing phenomenon was observed, and a Rabi splitting of 137 meV was extracted using COM. This work demonstrated that NPOM cavities can serve as highly controllable platforms for studying coupling in TMDs and highlighted the importance of nanogap engineering in modulating strong coupling strength. Progressing further, in 2020, Min Qiu’s group realized near-single exciton strong coupling using a gold nanoprism on a gold film cavity (Figure 12b) [43]. By tuning the dielectric spacer thickness, they reduced the number of excitons by two orders of magnitude (N≈2) compared to previous systems and observed Rabi splitting in PL. Silver nanocube-on-mirror structures, another typical NPOM geometry, have likewise been employed to achieve strong coupling with monolayer TMDCs [186,200]. For example, Han and colleagues constructed Ag nanocube Ag film nanocavities incorporating monolayer WS_2_ [186]. They demonstrated that the coupling state could be dynamically modulated by solvent infiltration into the nanogap, enabling active control over the interaction.

Beyond far-field optical detection, near-field approaches have emerged as powerful tools to directly probe strong coupling phenomena at the nanoscale, as conventional far-field optical methods can pick up additional emission from uncoupled excitons [99,121]. Douglas Natelson’s team fabricated hybrid structures consisting of plasmonic tunnel junctions integrated with few-layer TMDs (Figure 12c) [187]. Electroluminescence (EL) spectra under varying bias voltages, a near-field approach, revealed clear Rabi splitting exceeding 50 meV when plasmon and exciton modes resonated, demonstrating dynamic tuning of coupling strength. In the same year, Zhou et al. reported a gold nanotrench monolayer WSe_2_ hybrid structure (Figure 12d) [188], achieving strong coupling with Rabi splitting around 80 meV at room temperature. Using a fiber-optic pyramid near-field probe, they mapped plexciton emission at the nanoscale, revealing that emission originated from the lower polariton branch, localized within ~20 nm at the trench center, and exhibited pronounced in-plane polarization (Figure 12d). These findings provide direct experimental evidence of nanoscale plexciton emission properties and spatial distributions, advancing the fundamental understanding of interactions in TMDs plasmon hybrid systems.

#### 4.1.3. Other Plasmon TMDs Strong Coupling Systems

In this subsection, we turn our attention to non-particle plasmonic nanostructures and their strong coupling with 2D materials. Unlike individual nanoparticles or dimers, these extended or hybridized architectures including periodic arrays [170,189,190,201], gratings [54], metal insulator metal (MIM) systems [84,202], and thin-film-based cavities [203] offer distinct advantages, such as enhanced optical response, non-local lattice resonance mode, tunable dispersion relations, and reduced radiative losses. These features provide new pathways to exploration of the strong coupling with TMDs.

**Figure 13:**
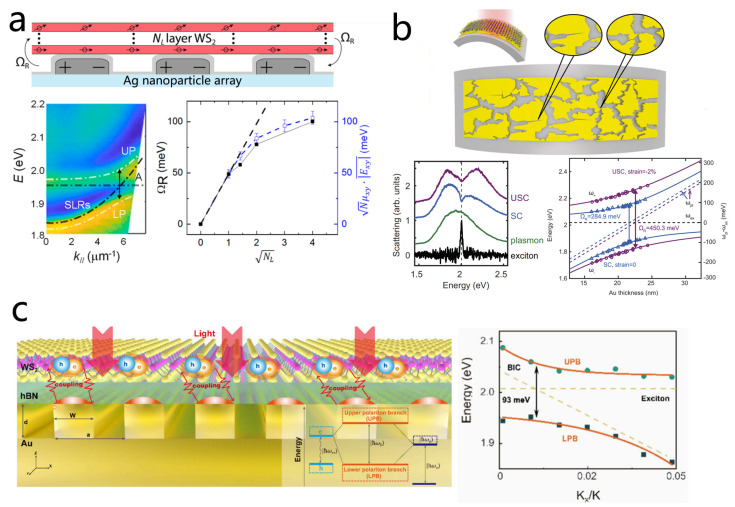
Strong coupling work of other structures with TMDs. (**a**) Top: Schematic of an open plasmonic cavity formed by a periodic array of metal nanoparticles. Bottom left: Dispersion measurement plot. Bottom right: Measured (black squares) and estimated (blue squares) Rabi energy as a function of the square root of the number of WSe_2_ layers. Adapted with permission from Ref. [189]. Copyright 2019 American Chemical Society. (**b**) Top: Schematic of a gold multi-singular metasurface with a dense array of nano-sized plasmonic gaps. Bottom left: Dark-field scattering spectra of the WS_2_ monolayer on polymer and the plasmonic metasurface uncoupled (green) and coupled (blue for SC and purple for USC) to WS_2_ excitons (arb. units). Bottom right: Dispersion plots of the measured dark-field scattering spectra. Adapted with permission from Ref. [170]. Copyright The Author(s) 2024. (**c**) Left: Schematic of strong coupling between a plasmonic BIC and a TMD exciton. The right panel depicts the strong coupling energy diagram when an exciton with energy ${\hbar \omega }_{ex}$ couples with a plasmonic BIC of energy ${\hbar \omega }_{ex}$. Strong coupling forms hybrid states (upper and lower polariton branches) separated by the Rabi splitting energy ${\hbar \omega }_{ex}$. Right: Dispersion relationship of polariton for the UPB and LPB fitted by the COM. Adapted with permission from Ref. [54]. Copyright 2025 American Chemical Society.

LSPRs offer strong field confinement and small mode volumes, enabling efficient coupling, but they inherently suffer from high losses [61,83,181,182]. In contrast, SLRs in periodic nanoparticle arrays provide an alternative: they exhibit much higher quality factors by suppressing radiative losses, extend the coherence time of Rabi oscillations, and feature tunable dispersive behavior governed by array periodicity [54,189,190,204]. These advantages make SLRs an ideal platform for realizing robust strong coupling. As shown in Figure 13a, Wang et al. investigated the strong coupling of multilayer WS_2_ with a silver nanoparticle array at room temperature [189]. By combining 1 to 16 layers of WS_2_ with an open plasmonic cavity supporting surface lattice resonances, they observed a clear anti-crossing phenomenon, with the Rabi energy increasing from 52 meV for a single layer to 100 meV for 16 layers. In 2022, Changzhi Gu’s team designed a coupled Au nanogroove array integrated with a WS_2_/hBN heterostructure. Here, strong coupling enabled the formation of polaritons with robust linear polarization at room temperature, which provides a potential solution to the long-standing challenge of valley coherence preservation [190].

Periodic metal arrays also serve as a fundamental platform for constructing plasmonic metamaterials [205,206]. When carefully designed, such arrays can endow materials with unconventional effective optical properties. Andrey E. Miroshnichenko and colleagues demonstrated strong coupling between TMDC monolayers and hybrid plasmonic metamaterial cavities [41], leveraging a novel catenary-shaped field enhancement mechanism. By carefully engineering the metamaterial geometry, they achieved pronounced local field confinement, enabling Rabi splittings of up to 320 meV at room temperature.

Bound states in the continuum (BICs) are nonradiating modes embedded within the continuum of radiative states [207,208,209,210], which is one of the unique modes supported by the plasmonic arrays. BICs remain perfectly confined even at energies where coupling to free-space radiation is allowed, effectively suppressing radiative losses. This unique property results in extremely high Q-factors and strong light confinement, thus greatly strengthening interactions, making them ideal for realizing strong coupling with excitons in 2D materials [211,212]. The resonance characteristics of BICs including frequency, polarization, and symmetry can also be precisely engineered through the geometry of the supporting structure, offering a versatile platform for tunable plexcitonic devices [213]. Luo et al. experimentally demonstrated a the strong coupling system between plasmonic BIC modes and WS_2_ [54], consisting of a one-dimensional gold grating atop a bottom gold film with monolayer WS_2_ transferred on top (Figure 13c). This architecture leverages the high-Q factor of BICs together with the subwavelength confinement of plasmonic nanogaps, optimizing the Q/V ratio critical for strong coupling. Using momentum-space imaging and PL measurements, the team observed a Rabi splitting of 93 meV, representing a significant step toward practical BIC-based plasmonic metamaterials.

Finally, we introduce a recent study that explores phenomena surpassing the strong coupling regime. When the strength of the interaction becomes comparable to the exciton energy itself, ultrastrong coupling occurs. In the ultrastrong coupling regime, the ground state of the system is altered and contains entanglement between photons and matter [28,214,215,216]. Yu Luo and colleagues reported room-temperature ultrastrong coupling in a WS_2_ monolayer integrated with a random multi-singular plasmonic metasurface deposited on a flexible polymer substrate (Figure 13b) [170]. The metasurface was engineered with a dense distribution of singular points, creating nanoscale plasmonic hotspots that enable coherent interaction of multiple excitons simultaneously. This design effectively concentrates the local electromagnetic field while increasing the number of excitons participating in the coupling. For a single WS_2_ layer, the normalized coupling strength reached 0.12, rising to 0.164 for a four-layer stack, demonstrating clear ultrastrong coupling. This work highlights the potential of multi-singular metasurfaces for enabling ultrastrong interactions in low-dimensional semiconductors, paving the way for advanced optoelectronic and quantum devices.

In summary, this chapter reviews recent progress in a strong coupling between surface plasmons and two-dimensional (2D) materials, with a particular focus on TMDs. Owing to their strong excitonic resonances, high binding energies, low losses, and excellent integrability, TMDs provide an ideal platform for realizing room-temperature coupling. A wide range of plasmonic architectures, from single nanoparticles (nanorods, nanocubes, bipyramids, and nanodisks) to nanogap-based systems (dimers, bowtie antennas, and NPOM structures), and further to extended platforms such as periodic arrays, gratings, metamaterials, and bound states in the continuum, successfully achieved strong coupling with TMDs. These studies demonstrate remarkable achievements, including Rabi splittings exceeding 200 meV, dynamic control of coupling strength, ultrastrong coupling, and even near-single-exciton interactions. Overall, the chapter highlights the unique advantages of 2D materials in strong coupling and underscores their great potential for next-generation nanophotonic, optoelectronic, and quantum devices.

## 5. Multimode Strong Coupling in Plasmonic Structure 2D Material Systems

### 5.1. Introduction to Multimode Strong Coupling

Conventional strong coupling typically involves the interaction between a single optical mode and a single type of quantum emitter, which can be well described by two coupled harmonic oscillators [48,85,121]. When additional optical modes or new quantum emitters are introduced, the system must instead be modeled as the coupling among their resonant modes of multiple oscillators [217,218]. Here, such systems are referred to as multimode strong coupling systems [219]. In multimode strong coupling systems, three or more hybrid quantum states are generated, and multiple distinct split peaks appear in the spectra, forming an anti-crossing dispersion curve with multiple branches [218,220,221].

The multimode strong coupling system offers several advantages over conventional counterparts, which only support two coherent states. First, multimode strong coupling systems open broader opportunities in practical applications in quantum optics, such as multi-entanglement [222], quantum computing [223], and quantum networks [224]. Second, in a multiple exciton system, multimode strong coupling provides additional energy dissipation channels and enhanced modulation capabilities, enabling active control of exciton plasmon exciton interactions through multiple excitonic states. This could establish highly efficient and ultrafast energy transfer pathways between different exciton states, which is particularly valuable for functional optoelectronic applications [221]. Moreover, multimode strong coupling can also introduce more degrees of freedom and multi-level structure for realizing controllable energy matching, wavelength conversion, and nonlinear optical modulation [217,225].

Two-dimensional materials, especially TMDs, serve as particularly attractive excitonic platforms for realizing multimode strong coupling [175,221,226]. In addition to their very large exciton binding energy, oscillator strength, and good stability, the various types of excitons contained in a single TMD such as the A exciton, B exciton, and trions also provide a natural and advantageous platform for realizing multimode strong coupling [175,218,226]. Moreover, 2D materials are easy to integrate with other types of exciton materials and plasmonic structures, such as nanoparticles coated with J-aggregates, etc. [221,225]. Therefore, TMDs are excellent exciton platforms for studying strong interactions involving multiple modes in plasmon systems.

Currently, three primary approaches are employed to achieve multimode strong coupling with TMDs: (i) coupling between a single plasmonic structure and multiple excitonic modes in TMDs [175,218,220,226]; (ii) hybrid multimode coupling involving plasmonic structures, TMDs, and J-aggregates [57,217,221,225]; and (iii) multimode systems combining plasmonic structures with optical microcavities and TMDs [180,215,227,228,229]. In the following, we first introduce the basic principles of three-mode strong coupling as an illustrative example and then review recent progress in multimode coupling systems based on TMDs.

### 5.2. Basic Principle of Multimode Strong Coupling

**Figure 14:**
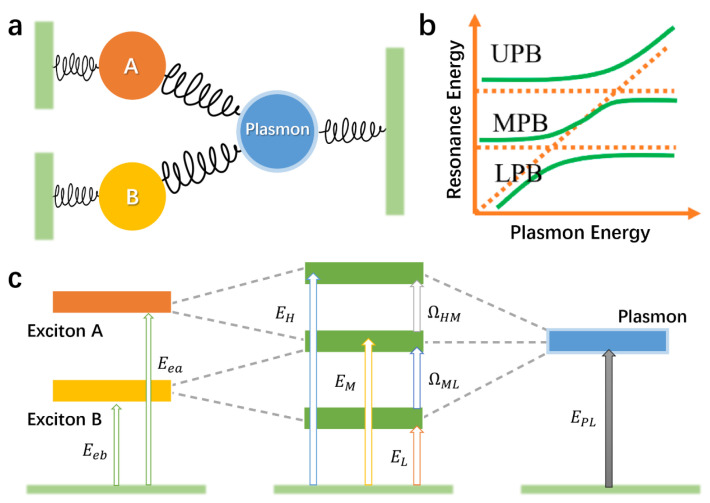
Schematic diagram of strong coupling of three modes. (**a**) Schematic diagram of the three-mode COM. (**b**) Double anti-crossing dispersion curves of multimode coupling. (**c**) Schematic diagram of the energy level splitting of plasmon and exciton multimode coupling.

In a multimode system, taking three-mode coupling as an illustrative example, the mechanism can be described by COM, as shown in Figure 14a. Here, a second exciton (B) is introduced in addition to the strong coupling between a single exciton (A) and a plasmon. Both excitons A and B interact with the plasmon, and there is no interaction between exciton A and exciton B. Due to the coupling interaction between the oscillators, the plasmon energy levels and the excitonic oscillator energies Epl, Eea, Eeb are renormalized and hybridized, as shown in Figure 14c. As a result, three hybridized polariton states emerge, accompanied by double Rabi splitting.

The equations of motion for the three oscillators are as follows [57,217]:

(9) $$\begin{array}{c} \ddot{x_{pl}}(t)+\gamma_{pl}\dot{x_{pl}}(t)+\omega_{pl}^{2}x_{pl}\left(t\right)+g_{a}\dot{x_{a}}(t)+g_{b}\dot{x_{b}}(t)=F_{pl}(t) \end{array}$$

(10) $$\ddot{x_{a}}(t)+\gamma_{a}\dot{x_{a}}(t)+\omega_{a}^{2}x_{a}\left(t\right)-g_{a}\dot{x_{pl}}(t)=F_{a}(t)$$

(11) $$\ddot{x_{b}}(t)+\gamma_{b}\dot{x_{b}}(t)+\omega_{b}^{2}x_{b}\left(t\right)-g_{b}\dot{x_{pl}}(t)=F_{b}(t)$$

Among them, $x_{pl}$, $x_{a}$, and $x_{b}$ represent the amplitudes of surface plasmon polaritons, exciton A oscillators, and exciton B oscillators, respectively. $\gamma_{pl}$, $\gamma_{a}$, and $\gamma_{b}$ are the damping rates of surface plasmon polaritons, exciton A, and exciton B. $\omega_{pl}$, $\omega_{a}$, and $\omega_{b}$ are the resonance frequencies of surface plasmon polaritons, exciton A, and exciton B. $g_{a}$ is the coupling rate between surface plasmon polaritons and exciton A, and $g_{b}$ is the coupling rate between surface plasmon polaritons and exciton B. $F_{pl},\;F_{a}$, and $F_{b}$ represent the driving forces generated by external sources. If both exciton A and exciton B are only driven by surface plasmon polariton oscillators; therefore, $F_{pl}(t)$=$F_{pl}e^{-i\omega t}$ at time ω is the electric field frequency, and $F_{a}(t)=0$, $F_{b}(t)=0$. Finally, $x_{pl}(t$), $x_{a}(t)$, and $x_{b}(t)$ can be derived from the above equations of motion. In the quasi-static limit, where the size of the structure is very small compared with the optical wavelength, the scattering cross-section is $\left(\frac{8\pi }{3}\right)*k^{4}{\left|F_{pl}x_{pl}\right|}^{2}$, and the wave vector of light is $k=\frac{\omega n}{c}$. Substituting $x_{pl}\left(t\right)$ into $\left(\frac{8\pi }{3}\right)*k^{4}{\left|F_{pl}x_{pl}\right|}^{2}$ and replacing the incident light frequency ω with the incident light energy E, and replacing the resonance frequency of plasmon, exciton A, and exciton B with the energy $E_{pl},\;E_{a},$ and $E_{b}$, the scattering cross-section formula can be obtained [58,85]:

(12) $$\begin{array}{c} \;\sigma_{scat}\left(E\right)=\left(\frac{8\pi }{3}\right)k^{4}{\left|F_{pl}x_{pl}\right|}^{2}\infty E^{4}{\left|\frac{ab}{ab\left(E^{2}-E_{pl}^{2}+iE\gamma_{pl}\right)-E^{2}g_{a}^{2}b-E^{2}g_{b}^{2}a}\right|}^{2} \end{array}$$

where $a=E^{2}-E_{a}^{2}+iE\gamma_{a}a$; $b=E^{2}-E_{b}^{2}+iE\gamma_{b}b$. Under the assumption of time-harmonic conditions, the eigen-equation of the system can be derived:

(13)  Epl−iγpl2gb2ga2gb2Eb−iγb20ga20Ea−iγa2αplαbαa=Eαplαbαa

Among them, $E_{pl}-i\frac{\gamma_{pl}}{2}$ and $U_{b}-i\frac{\gamma_{b}}{2}$ are the unperturbed complex energies of the optical modes in exciton A and exciton B, respectively, $E_{a}-i\frac{\gamma_{a}}{2}$ is the complex exciton energy, $g_{b}$ is the coupling constant between the optical modes, and $g_{a}$ is the exciton photon coupling potential. $a_{pl}$, $a_{b}$ and $a_{a}$ are the eigenvector components (Hopfield coefficients), ${\left|a_{pl}\right|}^{2},\;{\left|a_{b}\right|}^{2}$ and ${\left|a_{a}\right|}^{2}$ are the weighting efficiencies, and satisfy ${\left|a_{pl}\right|}^{2}+{\left|a_{b}\right|}^{2}+{\left|a_{a}\right|}^{2}=1$. The solution of the resonance secular determinant, where $E_{pl}-i\frac{\gamma_{pl}}{2}=E_{b}-i\frac{\gamma_{b}}{2}=E_{a}-i\frac{\gamma_{a}}{2}=0$, leads to E=0, $\pm \sqrt{\left(g_{a}^{2}+g_{b}^{2}\right)}$. From the above, there may be three modes, namely the central mode at the unperturbed energy and the two external modes shifted to higher and lower energies. The eigenvectors ψ of the UPB, MPB, and LPB modes are

(14) $$\begin{array}{c} \psi_{UPB}=\frac{1}{\sqrt{2}}\Phi_{pl}+\frac{g_{b}}{\sqrt{2}{\left({g_{a}}^{2}+{g_{b}}^{2}\right)}^{\frac{1}{2}}}\Phi_{b}+\frac{g_{b}}{\sqrt{2}{\left({g_{a}}^{2}+{g_{b}}^{2}\right)}^{\frac{1}{2}}}\Phi_{a} \end{array}$$

$$\begin{array}{c} \;\psi_{MPB}=\frac{△}{\sqrt{2}{\left({g_{a}}^{2}+{g_{b}}^{2}\right)}^{\frac{1}{2}}}\Phi_{b}-\frac{△}{\sqrt{2}{\left({g_{a}}^{2}+{g_{b}}^{2}\right)}^{\frac{1}{2}}}\Phi_{a}\; \end{array}$$

(15) $$\begin{array}{c} \;\psi_{UPB}=\frac{1}{\sqrt{2}}\Phi_{pl}-\frac{g_{b}}{\sqrt{2}{\left({g_{a}}^{2}+{g_{b}}^{2}\right)}^{\frac{1}{2}}}\Phi_{b}-\frac{g_{b}}{\sqrt{2}{\left({g_{a}}^{2}+{g_{b}}^{2}\right)}^{\frac{1}{2}}}\Phi_{a} \end{array}$$

Among them, $\Phi_{pl}$, $\Phi_{b}$, and $\Phi_{a}$ are the unperturbed basis functions. According to Equation (15), the eigenvector of the central mode of exciton A does not contain the component of the basis function $\Phi_{pl}$; for the central mode, the amplitude of the intracavity optical field that satisfies this condition is zero. All the weights of $\Phi_{pl}$ are equally distributed in the two outer components. When the plasmon energy is adjusted, we can plot the dispersion spectrum of the coupled system as shown in the Figure 14b. The dispersion relationship reveals three hybrid branches and two distinct anti-crossing behaviors. This image is an important basis for judging plasmon multimode strong coupling.

### 5.3. Advance in Multimode Strong Coupling with TMDs

#### 5.3.1. Strong Coupling Between Plasmons and Different Excitons in TMDs

The rich physics of TMDs gives rise to a variety of excited quasiparticles; in addition to multiple exciton states, this also includes charged trions, making TMDs highly advantageous for building multimode strong coupling platforms [175,220]. Due to their large binding energies (tens of meV), trions are stable even at room temperature [230]. Their populations can be effectively tuned via electrostatic gating, enabling reversible control of neutral and charged excitonic species [231]. Moreover, trions in monolayer WSe_2_ exhibit valley-selective optical transitions, adding an extra degree of freedom for valleytronic applications [232]. Collectively, these findings establish trions as highly interactive quasiparticles with stronger coupling, offering exciting opportunities for tunable polaritonic and valleytronic systems. Owing to these unique properties of trions, achieving their strong coupling with plasmons opens up new avenues for exploring interactions under extreme conditions.

**Figure 15:**
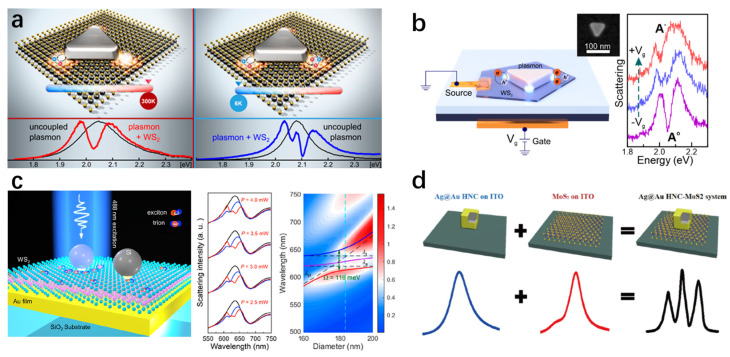
Strong coupling systems between plasmons and different excitons in TMDs. (**a**) Left: Schematic diagram of the plexcitonic system composed of silver nanopyramids and WS_2_ monolayers at room temperature (300 K) and the scattering spectra. Right: Schematic diagram of the multimode coupling system at low temperature (6 K) and the scattering spectra. Adapted with permission from Ref. [220]. Copyright 2018 American Chemical Society. (**b**) Left: Schematic diagram of the hybrid system under the action of an electric back gate. Right: Scattering spectrum of the WS_2_ monolayer under electrical control. Adapted with permission from Ref. [175]. Copyright 2020 American Chemical Society. (**c**) Left: coupling system in the Si/WS_2_/Au hybrid nanocavity. Middle: Scattering spectra (red solid lines) calculated for the Si/WS_2_/Au nanocavity under different power laser irradiations. Right: 2D scattering spectra calculated for the Si/WS_2_ nanocavity composed of nanoparticles with different diameters at the same laser power. Adapted with permission from Ref. [226]. Copyright 2022 American Chemical Society. (**d**) Single Ag@Au hollow nanocube schematic diagram of the energy level splitting caused by the strong coupling between the monolayer MoS_2_ composite structure. The upper figure is the model diagram of the composite structure, and the lower figure is the schematic diagram of double Rabi splitting. Adapted with permission from Ref. [218]. Copyright 2024 Optica Publishing Group.

As illustrated in Figure 15a, a hybrid system consisting of silver nanorods and a monolayer of WS_2_ was constructed by Cuadra et al. [220]. At room temperature (300 K), strong coupling between the LSPs of the nanorods and neutral excitons in WS_2_ was achieved, giving rise to plexciton. A key advancement of this work lies in extending the study to low temperature (6K), where temperature regulation enabled simultaneous strong coupling between plasmons, neutral excitons, and charged trions, thereby forming polaritons. This result demonstrates, for the first time, that trions can also participate in multimode strong coupling. Building upon this system, the authors further introduced electrical gating to actively tune the oscillator strengths of neutral and charged excitons in the WS_2_ monolayer [175]. As shown in Figure 15b, electrical control enabled reversible modulation of the strong coupling strength. This finding provides a pathway toward achieving room-temperature tunable multimode polariton devices.

Subsequently, Liu et al. proposed an alternative approach based on optical regulation [226]. By constructing a dielectric metal hybrid nanocavity (Si/WS_2_/Au, Figure 15c), they demonstrated that external laser excitation could dynamically modulate the coupling. The laser power was shown to directly influence the coupling strength and drive the formation of multimode coupled states. Compared to temperature or electrical control, optical regulation offers a faster response and avoids thermal damage to fragile 2D materials.

Monolayer MoS_2_ serves as an ideal platform for double exciton coupling, which is capable of forming two stable excitonic states (A and B excitons) at room temperature. These unique excitonic states have been identified as direct excitonic transitions at the K-point of the Brillouin zone [233]. The energy difference is due to the spin-orbit splitting of the valence band, which results in two resonances, the A and B excitons. You et al. investigated a plexcitonic system involving these excitonic states. They observed simultaneous strong coupling of A and B excitons in monolayer MoS_2_ with Ag@Au hollow nanocube [218]. More importantly, they achieved the demonstration of characteristic double Rabi splitting in this bi strong coupling system (Figure 15d).

#### 5.3.2. Plasmonic Nanoparticles TMDs J-Aggregates Strong Coupling Systems

Beyond excitonic states intrinsic to 2D materials, additional excitonic systems can be incorporated to construct biexciton strong coupling platforms. A representative example involves the combination of TMD monolayers with J-aggregates, organic molecular assemblies that exhibit large oscillator strengths and narrow linewidths [16,37,234]. Compared with exploiting multiple excitonic states within a single two-dimensional material, constructing multimode systems from different types of excitonic materials offers greater flexibility, as various excitonic components can be combined according to specific requirements. On the other hand, however, the integration of these distinct excitonic materials poses new challenges.

**Figure 16:**
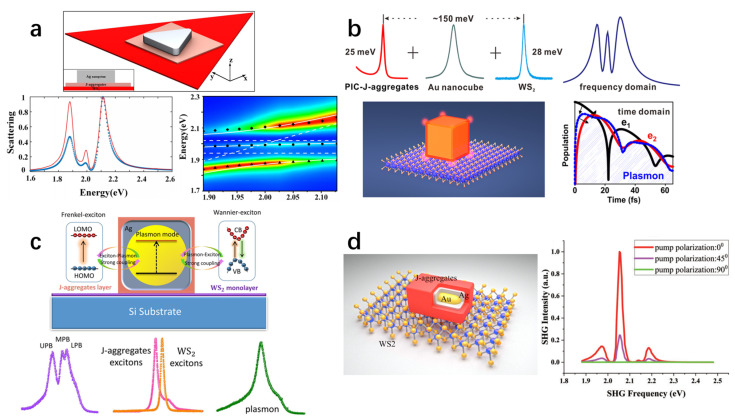
Plasmonic nanoparticles TMDs J-aggregates strong coupling systems. (**a**)**.** Upper panel: schematic diagram of the Ag nanoprism J-aggregates WS_2_ nanostructure. Lower left: scattering spectra of the multimode coupling system. Lower right: scattering spectra of the multimode coupling system as a function of plasmon energy. Adapted with permission from Ref. [57]. Copyright 2019 Optical Society of America. (**b**) Upper panel: three-peak spectrogram of hybrid nanoparticle (HNP)/WS_2_. Lower left panel: Schematic diagram of the AuNC@J-aggregates HNP structure located on the WS_2_ monolayer. Lower right: time-dependent populations of two emitters in the plasmonic cavity. Adapted with permission from Ref. [221]. Copyright 2021 The Authors. (**c**) Upper panel: Quantum emitter nanoantenna structure capable of simultaneously exchanging energy with both Frenkel excitons and Wannier excitons. The newly formed bi hybrid state can possess the characteristics of both quantum emitters. The measured spectra of the uncoupled components (lower rightmost panel) and the multimode strongly coupled nanosystem (lower leftmost panel) exhibit distinct double Rabi splitting features. Adapted with permission from Ref. [225]. Copyright 2023 the author(s). (**d**) Left: coupled nanostructure composed of a monolayer WS_2_ and a single Au@Ag nanorod covered by J-aggregates. Right: calculated SHG scattering spectra of a single cuboid Au@Ag nanorod coated with a monolayer of WS_2_ at different pump polarization angles. Adapted with permission from Ref. [217]. Copyright 2023 Optica Publishing Group.

Jiang et al. theoretically investigated a hybrid structure consisting of silver nanorods, J-aggregates, and monolayer WS_2_ (Figure 16a) [57]. FDTD simulations and COM analysis revealed that this system supports simultaneous three-mode coupling between localized plasmons, J-aggregate excitons, and WS_2_ excitons. Moreover, the coupling strength was shown to be tunable by adjusting temperature and J-aggregate concentration, offering a practical route for active control.

Based on this theory, Zhang et al. shifted the experimental focus from single excitons to a biexciton strong coupling system at room temperature [221]. As shown in Figure 16b, they found that J-aggregate excitons and WS_2_ excitons with different energies could simultaneously have strong coupling with gold nanocubes. They also observed that strong coupling behavior could still be seen at room temperature, even when the energy difference between the two excitons was about 5 times the spectral linewidth of each. In this paper, further exploration was carried out in plasmon-assisted coherent energy transfer, and it was demonstrated that active control of the energy transfer process can be achieved by regulating the plasmon resonance energy, loss rate, and coupling strength.

On this basis, a novel Au@Ag nanocube architecture was developed (Figure 16c) [225], enabling fine-tuned control over bi interactions. Systematic studies revealed mode competition between the two exciton modes (J-aggregates and WS_2_) and plasmons, with an optimal J-aggregate thickness yielding the strongest double Rabi splitting. Hopfield coefficient analysis further showed that J-aggregates and WS_2_ excitons contribute dominantly to the intermediate polariton branch, while plasmonic contributions are relatively minor.

The work of Li et al. is also dedicated to the research of the biexciton strong coupling system (Figure 16d) [217]. However, the most significant innovation point of this research lies in the combination of nonlinear optical effects, second harmonic generation (SHG), with biexciton strong coupling. The usual research object linear scattering spectrum is extended to the SHG scattering spectrum. It is found that in the biexciton strong coupling system, SHG exhibits a triple-polariton branch similar to the linear scattering spectrum, as shown in the right of Figure 16d.

#### 5.3.3. Plasmonic Nanoparticle Microcavity TMDs Strong Coupling System

**Figure 17:**
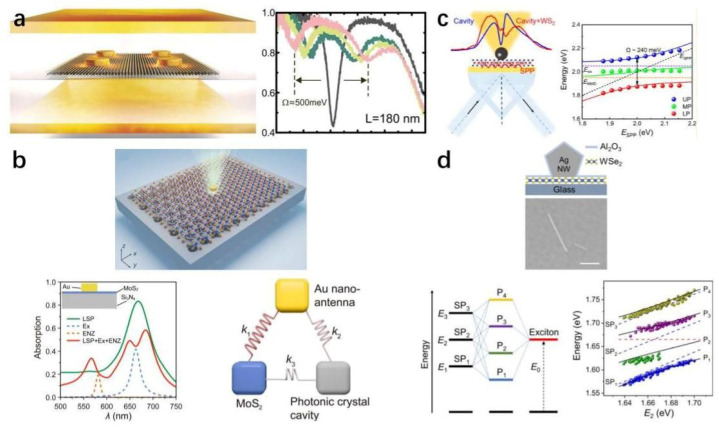
Plasmonic Nanoparticle Microcavity TMDs Strong Coupling System. (**a**) Left: Schematic diagram of a microcavity coupled with a square array of gold nanodisks. Right: Reflection measurements of the coupling system, showing a Rabi splitting of 500 meV. Adapted with permission from Ref. [227]. Copyright 2018 American Chemical Society. (**b**) Top: Schematic diagram of a single layer of MoS_2_ coupled with an AuNA-PC cavity hybrid resonator. Lower left: Absorption curves of the LSP mode, dipole (Ex) mode, plasmon-enhanced (ENZ) mode, and their coupled modes. Lower right: three-coupled oscillator model of the hybrid system. Adapted with permission from Ref. [215]. Copyright 2021 American Physical Society. (**c**) Left: Schematic diagram of Si/WS_2_/Au nanocavity coupling structure. Right: UP, MP, and LP branches (solid symbols) extracted from the scattering spectra. Adapted with permission from Ref. [228]. Copyright 2021 American Chemical Society. (**d**) Top: Cross-sectional schematic diagram and optical microscope image of AgNW on a single layer of WS2. Lower left: Schematic diagram of four composite exciton states resulting from strong coupling between three plasmon modes and one exciton state. Lower right: Energies of the four-hybrid branch as a function of the plasmon energy. Adapted with permission from Ref. [229]. Copyright 2023 the author(s).

As discussed in the introduction, multimode strong coupling can be realized by introducing additional optical modes into the system. Microcavities offer additional confined photonic modes [235,236], which can couple simultaneously with plasmons and excitons, thereby forming multimode polaritonic states with enhanced interaction strengths. Microcavity modes offer unique advantages, including high quality factors, low optical losses, and well-established fabrication techniques. Integrating microcavities with plasmonic structures thus opens up new avenues for the design of multimode hybrid systems, combining the long photon lifetime of microcavities with the strong field confinement of plasmonic modes. A pioneering experiment integrated Au nanoparticle arrays with a metal Fabry Pérot microcavity embedding WS_2_ monolayers [227], as shown in Figure 17a. This configuration enabled simultaneous coupling of cavity photons, plasmon resonances, and TMD excitons, yielding a Rabi splitting up to 500 meV far exceeding previous reports [227]. The scheme relaxes the need for high exciton densities and opens new possibilities for observing strong coupling in diverse material systems. The work of Li et al. further developed and verified this scheme [180], successfully strongly coupled the optical microcavity mode, the plasmons in Ag nanoparticles, and the A exciton of WS_2_ monolayer. They compared the coupling strengths of Ag-WS_2_ heterostructures with and without an optical microcavity. Embedding the plexcitonic system into the cavity produced a large Rabi splitting of 300 meV, confirming the crucial role of microcavities in enhancing interactions. The generated quasiparticle with part-plasmon, part-exciton, and part-light is analyzed with Hopfield coefficients that are calculated by the three-coupled oscillator model. Liu et al. further advanced this design by fabricating an Au nanoparticle photonic crystal (AuNA-PC) hybrid cavity (Figure 17b) [215]. The resulting system exhibited an even higher coupling strength of 460 meV, along with a complex four-body polaritonic mechanism involving cavity photons, plasmons, and multiple excitons. This work underscores the potential of multimode coupling to generate new quasiparticle states with additional degrees of freedom.

In recent years, dielectric nanoparticles supporting Mie resonances have also been employed to achieve resonant coupling with TMD monolayers. Photon exciton coupling approaching the strong coupling regime has been demonstrated using a silicon (Si) nanoparticle coupled to a WS_2_ monolayer [237,238]. In this system, the energy transfer is mediated by the magnetic dipole (MD) resonance of the Si nanoparticle, and the coupling strength can be tuned by modifying the surrounding environment. The research by Deng et al. used Si nanoparticle Au thin films as a new dielectric metal hybrid nanocavity structure [228], combining the surface plasmon polaritons on the gold film surface and the magnetic dipole resonance mode of the silicon sphere, as shown in Figure 17c Left. Through three-mode interactions among magnetic dipoles, surface plasmons, and WS_2_ excitons, a Rabi splitting of 240 meV was obtained, highlighting the cooperative enhancement achieved in multi-component coupling systems.

Lastly, the various different modes supported by the same plasmonic structure can also form strong multimode coupling with TMD excitons. Nie et al. reported strong coupling between a single silver nanowire and a WS_2_ monolayer, where three distinct plasmon modes simultaneously coupled to excitons, forming four plexcitonic branches (Figure 17d) [229]. The degree of coupling was tunable via excitation efficiency, marking the realization of such multimode strong interactions at room temperature.

Overall, researchers have successfully realized multimode strong coupling between excitons in two-dimensional materials and plasmons across diverse systems, enriching the physical picture of hybrid states. As the understanding of these systems advances, they offer a versatile platform for investigating complex interactions, paving the way for quantum optics applications.

## 6. Summary and Outlook

Remarkable progress has been made in understanding and realizing strong coupling between plasmonic nanostructures and low-dimensional materials, especially QDs and TMDs. Various plasmonic architectures including nanoparticles [181], nanodimers [59], NPOM structures [185], and periodic arrays [227] have been exploited to reduce mode volume, enhance local fields, and achieve strong interactions with low-dimensional materials at ambient conditions. These efforts have led to significant achievements, such as few-exciton and even near-single-exciton strong coupling [59], multimode strong coupling [228], ultrastrong coupling [170], and dynamic coupling control through electrical [175], optical [226], or environmental modulation [83]. Applications of plasmon–exciton strong coupling systems have already been demonstrated in several areas. For instance, they enable low-threshold nanolasers by exciton polariton condensation [239]. Bose Einstein condensation of exciton polaritons has also been observed [240], providing a unique platform for studying macroscopic quantum phenomena at room temperature. In addition, strong coupling has been used to build a new strategy for high-brightness, strongly circularly polarized OLEDs [241]. Despite these promising demonstrations, most of these applications remain at the proof-of-concept stage. Achieving practical photonic devices will require overcoming key challenges. The inherent losses of metallic nanostructures limit the coherence time of polaritons [121]. Moreover, translating these phenomena into scalable, device-ready architectures requires progress in nanofabrication, material integration, and deterministic positioning of excitons relative to hotspots [59,185].

Looking forward, several concrete directions may provide solutions to the current challenges in plasmon–exciton strong coupling. The first is loss reduction and stability improvement. Integrating alternative materials and platforms, such as sodium-based plasmonic structures [242], low-loss plasmonic modes [121], or hybrid plasmon–dielectric nanostructures [53], together with improved fabrication reproducibility, will be essential for building stable, long-term coherence plexcitonic platforms. The second is deterministic emitter–cavity integration. Future advances in quantum dot position, deterministic transfer of 2D materials, or template-assisted assembly strategies (e.g., DNA origami or nanopatterned substrates) may enable reliable positioning at the single-emitter level [15,55,243]. The third is exploring novel optical properties that emerge in strong coupling systems, such as nonlinear responses [81], chiral optical effects [48,91], and polarization-dependent phenomena [80]. This will deepen our understanding of light–matter interactions and provide new design principles for advanced nanophotonic and quantum devices.

In conclusion, strong coupling in low-dimensional materials represents not only fertile ground for exploring fundamental physics but also a promising route toward next-generation nanophotonic and quantum technologies.

## Figures and Tables

**Table 1:** Comparison of dye molecules, J-aggregates, QDs, and TMDs.

Characteristics	Dye Molecules [16,51,56]	J-Aggregates [48,57,58]	QDs [39,59,60]	2D Materials (TMDs) [40,61,62]
Oscillator strength	Low, limited by molecular dipole moment	Moderate, enhanced by collective excitonic effects	High, strong excitonic resonances, size-tunable	High, large exciton binding energy and oscillator strength
Stability	Prone to photobleaching and chemical degradation	Improved over single dyes but still susceptible to degradation	High photostability, resistant to photobleaching	Excellent chemical and thermal stability
Tunability	Dependent on molecular structure	Tunable via aggregate size and composition	Highly tunable via size, shape, and composition	Bandgap tunable via layer number, temperature, voltage, and magnetic field
Scalability	Poor photostability limits long-term use	Dynamic assembly prevents integration	Scalable synthesis via colloidal methods	Mechanical exfoliation limits scalability, improving CVD
Loss	40–700 meV	50–170 meV	50–110 meV	20–60 meV
Key challenges	Long-term stability and limited tunability	Long-term stability and structural variability	Surface defect control and toxicity concerns	Defect control, layer uniformity, and integration challenges

**Table 2:** Summary of strong coupling works between different plasmonic structures and QDs.

Structure	QD	QD Number	Rabi Splitting	Spectra
Silver nanoshells [124]	CdSe/Zns	Collective	160 meV	PL
Gold nanorods [125]	Pbs	Collective	231 meV	Absorption
Gold nanorod [15]	CdSe/ZnS	1	234 meV	Scattering
Gold dimer [26]	CdSe/CdS	1	185 meV	Scattering/PL
Silver bowtie [39]	CdSe/Zns	1–3	120 meV	Scattering/PL/EELS
Silver bowtie [99]	CdSe/ZnS	4–10	85 meV	EELS
Silver bowtie [59]	CdSe/ZnS	1–3	71.7 meV	Scattering/PL
One-dimensional photonic crystal Dimer [126]	CdSe/ZnS	1	170 meV	PL
Nanoparticle-on-mirror [28]	CdSe/CdS	1	200 meV	PL
Tip-on-film [127]	CdSe/ZnS	1	163 meV	Tip-enhanced photoluminescence
Silver film [111]	CdSe	Collective	112 meV	Attenuated total reflection
Gold nanohole array [128]	CdSe	Collective	220 meV	Absorption
Silver nanoparticle array [116]	CdSe/ZnS	Collective	110 meV	PL

**Table 3:** Summary of strong coupling works between different plasmonic structures and TMDs.

Structure	TMDs	Layer Number	Rabi Splitting	Spectra
Silver nanorod [83]	WSe_2_	Monolayer	49.5 meV	Scattering
Gold nanorod [181]	WS_2_	Monolayer	91–133 meV	Scattering
Au@Ag nanocube [61]	WS_2_	Monolayer	60.1 meV	Scattering/PL
Gold bipyramid [182]	WSe_2_	Mono-multilayer	83–105 meV	Scattering
Ag nanobipyramid-on-mirror [40]	WSe_2_/WS_2_	Monolayer	35/53 meV	Scattering/reflection/PL
Gold nanodisk [62]	WS_2_	Mono-multilayer	108/175 meV	Scattering/reflection
Gold dimer antenna [176]	WS_2_	Monolayer	115.2–128.6 meV	Scattering/PL
Gold bowtie antenna [183]	MoSe_2_	1–8 layer	80–110 meV	Scattering
Bowtie/Bloch surface waves hybrid cavity [184]	WSe_2_	Monolayer	186 meV	Scattering/reflection
NPOM [185]	WSe_2_	Multilayer	137 meV	Scattering
Au nanoprism-on-film [43]	WS_2_	Monolayer	163 meV	Scattering/PL
Ag nanocube-on-mirror [186]	WS_2_	Monolayer	145 meV	Scattering
Plasmonic tunnel junction [187]	WSe_2_	Few-layer	>50 meV	Electroluminescence
Gold nanotrench [188]	WSe_2_	Monolayer	80 meV	Near-field PL mapping
Ag nanoparticle array [189]	WS_2_	1–16 layer	52–100 meV	Scattering/reflection
Au nanogroove array [190]	WS_2_/HBN	Multilayer	42–65 meV	Scattering
Plasmonic metamaterial [41]	MoSe_2_/WSe_2_	Monolayer	77.86–320 meV	Scattering/reflection
Plasmonic grating [54]	WS_2_	Monolayer	93 meV	PL
Multi-singular metasurface [170]	WS_2_	1–4 layer	165.9–240.4 meV	Scattering

## Data Availability

Not applicable.
